# Congenital heart disease: types, pathophysiology, diagnosis, and treatment options

**DOI:** 10.1002/mco2.631

**Published:** 2024-07-05

**Authors:** Xiao Meng, Ming Song, Kai Zhang, Weida Lu, Yunyi Li, Cheng Zhang, Yun Zhang

**Affiliations:** ^1^ Department of Cardiology State Key Laboratory for Innovation and Transformation of Luobing Theory Qilu Hospital of Shandong University Jinan China; ^2^ Key Laboratory of Cardiovascular Remodeling and Function Research Chinese Ministry of Education, Chinese National Health Commission and Chinese Academy of Medical Sciences, Qilu Hospital of Shandong University Jinan China; ^3^ Shandong Key Laboratory of Cardiovascular Proteomics and Department of Geriatric Medicine Qilu Hospital of Shandong University Jinan China

**Keywords:** congenital heart disease, diagnosis, etiology, pathophysiology, treatment

## Abstract

Congenital heart disease (CHD) is a structural abnormality of the heart and/or great vessels and patients with CHD are at an increased risks of various morbidities throughout their lives and reduced long‐term survival. Eventually, CHD may result in various complications including heart failure, arrhythmias, stroke, pneumonia, and sudden death. Unfortunately, the exact etiology and pathophysiology of some CHD remain unclear. Although the quality of life and prognosis of patients with CHD have significantly improved following technological advancement, the influence of CHD is lifelong, especially in patients with complicated CHD. Thus, the management of CHD remains a challenge due to its high prevalence. Finally, there are some disagreements on CHD among international guidelines. In this review, we provide an update of the pathophysiology, diagnosis, and treatment in most common type of CHD, including patent foramen ovale, atrial septal defect, ventricular septal defect, atrioventricular septal defect, patent ductus arteriosus, coarctation of the aorta, transposition of the great arteries, congenitally corrected transposition of the great arteries, coronary anomalies, left and right ventricular outflow tract obstruction, tetralogy of Fallot and Ebstein anomaly. In particular, we focus on what is known and what is unknown in these areas, aiming to improve the current understanding of various types of CHD.

## Graphical abstract

In this review, we provide an update of the pathophysiology, diagnosis, and treatment in most common type of CHD, including patent foramen ovale, atrial septal defect, ventricular septal defect, atrioventricular septal defect, patent ductus arteriosus, coarctation of the aorta, transposition of the great arteries, corrected transposition of the great arteries, coronary anomalies, left and right ventricular outflow tract obstruction, tetralogy of Fallot and Ebstein anomaly.

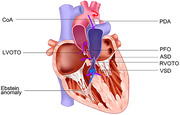

## 1 INTRODUCTION

Congenital heart disease (CHD) is a structural abnormality of the heart and/or great vessels occurring at birth that results in a series of short‐ and long‐term adverse sequelae.^1^ It accounts for 3% of neonatal death and 46% of death from all congenital malformations and is the leading cause of neonatal mortality.^2^, ^3^ There is a marked heterogeneity of CHD in different geographic regions worldwide. The average prevalence of CHD in Asia is significantly higher than that in Africa, resulting in a higher burden of medical care in Asia.^2^ In general, CHD can be classified as mild, moderate, or severe, based on the underlying anatomy and hemodynamic impact.^4^, ^5^ Patent foramen ovale (PFO), atrial septal defect (ASD), ventricular septal defect (VSD), and patent ductus arteriosus (PDA) are the most frequent types of CHD, accounting for the most of CHD cases.

The etiology of CHD is complicated and the underlying pathogenesis remains unclear in approximately 50% of patients with CHD. However, several environmental and genetic factors are found to be involved in the pathogenesis of CHD. Smoking is considered teratogenic, and smoking‐induced hemodynamic disorders may facilitate morphological or functional abnormalities in the fetal cardiovascular system.^6^ Carbon monoxide and nicotine can be harmful to fetal cardiac development.^6^ A meta‐analysis of observational studies involving a total of 137,574 patients with CHD found that maternal active and passive smoking as well as paternal active smoking increase the risk of CHD in offspring, suggesting that the cessation of parental smoking during peri‐pregnancy is a priority for the prevention of CHD. Moreover, subgroup analyses revealed that active maternal smoking rather than passive smoking is associated with the risk of ASD and right ventricular outflow tract obstruction (RVOTO).^6^ Another meta‐analysis revealed a nonlinear dose‐response relationship between parental alcohol consumption and the risk of CHD in offspring, with the risk of CHD in offspring gradually increasing as parental alcohol consumption increases.^7^ Therefore, reducing the preconception and gestational parental alcohol consumption may help prevent CHD in offspring.

The heart is one of the first organs to develop during embryogenesis. The development of the embryonic heart is a precisely controlled process. Changes at the gene level may affect cell proliferation, differentiation, and migration, which are critical processes for embryonic development. Gene dysregulation, including abnormalities in genes encoding transcription factors, signaling pathways, and chromatin modifiers, can interfere with the specification and differentiation of cells and offset the normal development of tissues and organs, resulting in birth defects, including CHD.^8^, ^9^ Approximately 400 gene abnormalities are associated with the pathogenesis of CHD and 10−30% of structural CHD cases are due to genetic mutations.^4^, ^9^ CHD may be genetically heterogeneous, as the recurrence risk of CHD in offspring is higher when the mother is affected than that when the father is affected.^4^ Therefore, further epigenetic investigation is necessary to assess the association between the genetic architecture and CHD pathogenesis. Targeting genes may help identify potential biomarkers that serve as therapeutic targets to decrease the risk of CHD development.

Compared with the general population, patients with CHD have an increased risk of various morbidities throughout their lives and reduced long‐term survival.^10^ CHD may result in various complication, such as heart failure, arrhythmias, stroke, pneumonia, hemorrhage, and sudden death.^10^ The prevalence of sudden cardiac death (SCD) in patients with CHD is approximately 0.28–2.7% per year, which is 20–30‐fold higher than that in the general population.^11^, ^12^ Moreover, mortality is considerably higher among patients with complex CHD. Due to improvements in cardiovascular diagnostics and treatment, most types of CHD, including PFO, ASD, VSD, and PDA, can be treated using transcatheter and surgical approaches. Improved rates of overall survival and outcomes in patients with CHD have been reported in recent decades. Consequently, most patients born with CHD may now survive into adulthood and grow well.^1^ Nonetheless, some grown‐up patients with CHD (GUCH) still require long‐term medical care, especially patients with GUCH who have undergone complicated surgery.^13^ In some developing countries and regions, access to efficient treatments for complex CHD remains limited.

Despite advances in the medical domain, some problems of CHD remain unclear. The management of CHD is challenging due to its overall prevalence. There exit controversies on the management of CHD among established international guidelines.^5^ For instance, there are substantive differences between the American Heart Association (AHA)/American College of Cardiology (ACC) guidelines and European Society of Cardiology (ESC) guidelines for the management CHD. Therefore, an updated overview of the pathophysiology, diagnosis, and treatment of CHD is necessary. This extensive review provides an update of the pathophysiology, diagnosis, and treatment of most common types of CHD, focusing on what is known and what is unknown, to improve the current understanding of CHD.

## 2 PATENT FORAMEN OVALE

### 2.1 Pathophysiology of PFO

PFO as the most common CHD is found in almost one quarter of adults.^14^ Initiated around 4 weeks of gestation, the septum primum emerges and perforations appear within the growing septum primum concurrently, leading to the formation of the foramen secundum upon their fusion. Simultaneously, a membrane originates from the ventrocranial atrial wall, giving rise to the septum secundum, which gradually enlarges and overlaps with the foramen secundum, eventually creating an oval‐shaped aperture recognized as the foramen ovale.^14^, ^15^ During the fetal lifespan, the foramen ovale serves a physiological communication between the right and left atria, that allows maternally oxygenated blood to pass through, thus bypassing pulmonary circulation.^14^ After birth, the neonatal lungs begin to participate in oxygen exchange and recruitment of the pulmonary vasculature results in a reversed right‐to‐left atrial pressure gradient and eventually closure of the foramen ovale in the first 2 years.^16^ However, when this closing phenomenon fails, PFO persists throughout life.

PFO is heterogeneous in size, ranging from 1 to 19 mm, with an average of 4.9 mm in diameter.^17^ Previous studies reported that the mean PFO size increases per decade of life, with 3.4 mm in the first decade and 5.8 mm in the tenth decade of life.^14^ On the other hand, the prevalence of PFO decreases gradually with increasing age, from 34% in the first three decades to 20% in the ninth decade.^18^ However, there is no sex predominance and race‐ethnic variation in the prevalence of PFO.^14^ In a cryptogenic stroke study (PICSS—the PFO in Cryptogenic Stroke Study) of 630 patients with ischemic stroke, the incidence of PFO did not differ among Caucasians, Blacks, and Hispanics (34, 31, and 37%, respectively).^19^ Interestingly, a large PFO was more prevalent among Caucasians and Hispanics than among Blacks.^19^ However, the study did not include Asians. Kuramoto et al.^20^ analyzed 103 Japanese autopsy cases and showed that PFO incidence was 13.6% in the cohort, which was lower than reported in prior studies. It is an autopsy study with a small cases, that may not represent the general Japanese population. Therefore, large international studies of PFO including different ethnic groups are necessary to clarify whether there is a racial and regional disparity.

When PFO is open, it represents a door‐shaped channel rather than a real hole‐like aperture, permitting blood to flow through the PFO and forming right‐to‐left (R‐L) shunt. However, a R‐L shunt is not present in normal subjects because right atrial pressure is always below than that in the left. In contrast, PFO permits intracardiac shunting when right atrial pressure exceeds left atrial pressure, such as in pulmonary hypertension and during Valsalva maneuver.^21^ It has been estimated that PFO accounts for up to 95% of R‐L shunts.^21^ Less common etiologies for R‐L shunt include pulmonary arteriovenous malformations and ASD.^21^

**FIGURE 1:**
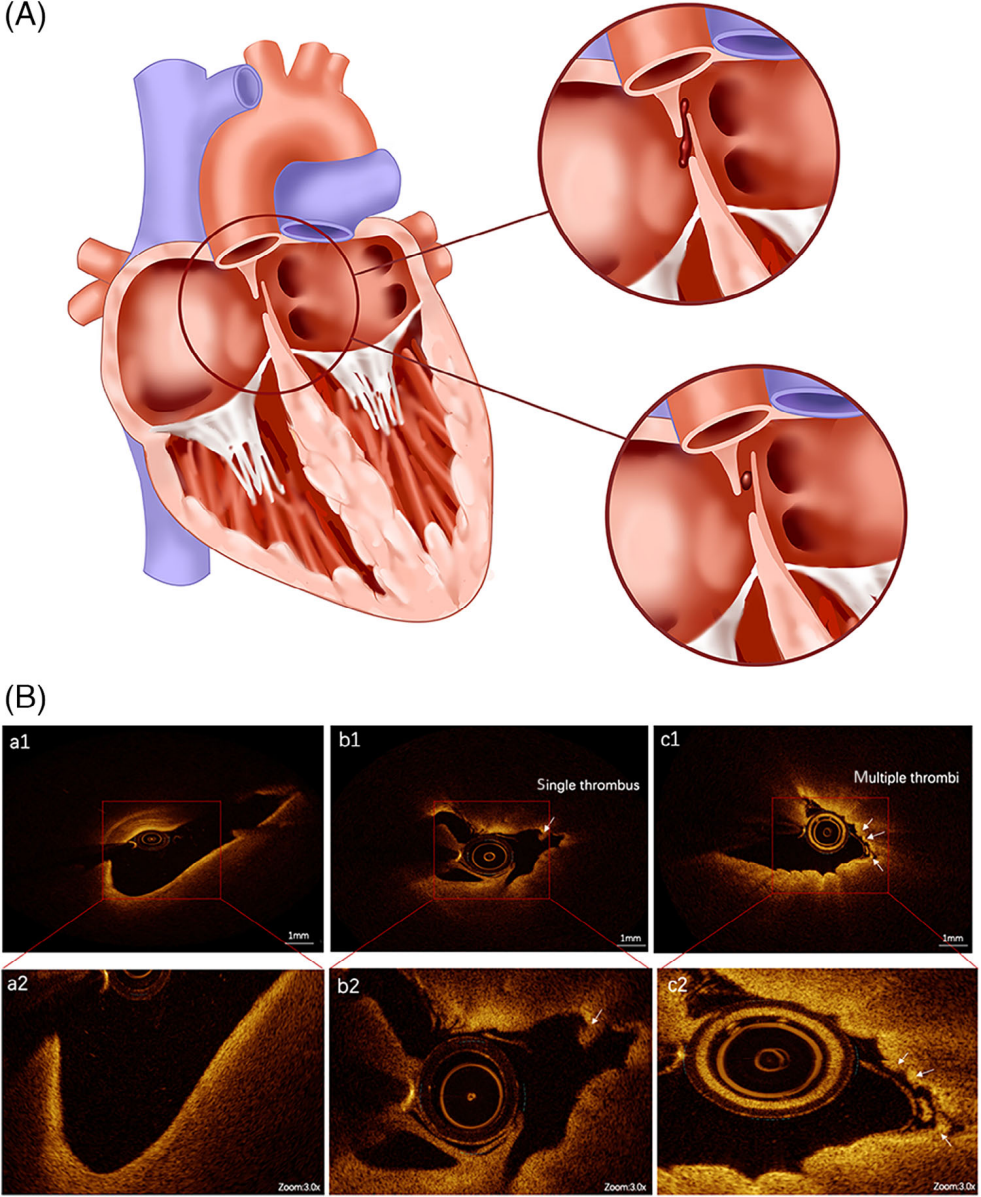
The underlying mechanism of patent foramen ovale (PFO)‐mediated paradoxical embolism. On the one hand, an open PFO acts as a potential pathway and predisposes embolus passage; on the other hand, an “in situ” thrombus may form in the PFO channel. (A) In situ thrombus within PFO assessed by high‐resolution optical coherence tomography (OCT). (B) Endocardium of PFO was smooth without thrombus (a1 and a2); an in situ thrombus was found on the endocardial surface of PFO (arrow) (b1 and b2); multiple in situ thrombi were found on the endocardial surface of PFO (arrows) (c1 and c2).

In most individuals, patients with PFO are completely asymptomatic and PFO per se has no pathogenicity. However, PFO is correlated with many pathological conditions, such as cerebrovascular disease, migraine headache, decompression illness, platypnea‐orthodeoxia syndrome, and obstructive sleep apnoea.^14^ Importantly, PFO may act as a potential pathway of R‐L shunt facilitating the embolus originating from the vein to reach the arterial system (Figure 1), which predisposes individuals to paradoxical embolic events with adverse outcomes, such as stroke, myocardial infarction, and peripheral ischemia. In this review, we summarize current evidence from clinical studies of PFO‐mediated paradoxical embolism in various pathological conditions.

### 2.2 PFO and paradoxical embolism

Paradoxical embolism was first reported by Cohnheim in 1877, referring to the occlusion of an artery due to the passage of venous embolic materials originating from the veins into the arterial circulation without passing through the lung circulation, which ordinarily acts as a filter of embolic materials.^22^ However, paradoxical embolism is uncommon and accounts for less than 2% of all arterial embolic events.^23^ As the most common intracardiac defect, PFO has been implicated as a potential mechanism for paradoxical embolism, permitting the venous embolus arrive into arterial circulation through PFO.^22^ In some physiological (such as coughing) and pathological conditions (such as pulmonary hypertension), increased right atrial pressure may induce R‐L shunt, enhancing the opportunity of paradoxical embolization in target organ.^14^ The main clinical manifestation of PFO‐mediated paradoxical embolism is thrombotic occlusion of the arterial system, and the sequelae are highly dependent on the site of embolization.

Identifying a source of emboli in PFO‐mediated paradoxical embolism is important. Deep venous thrombosis (DVT) is a potential source of emboli across the PFO. Individuals with PFO and suspected paradoxical embolism should be asked about their history of DVT, and DVT should be carefully assessed by physical examination and laboratory test. In a few cases, a thrombus lying in the tunnel of a PFO was demonstrated by echocardiography, confirming PFO as potential pathway of paradoxical embolism.^24^ In most cases, however, no thrombus straddling the PFO was found despite the application of transesophageal echocardiography (TEE). In recent years, with increasing clinical applications of optical coherence tomography (OCT), an “in situ” thrombus in the PFO channel has been found to be a possible mechanism of paradoxical embolism.^25^ Yan and Li^26^ assessed the microstructure of PFO with OCT in 11 patients with stroke and seven patients with migraine headache without stroke, and found multiple in situ thrombi on the endocardial surface in all patients with stroke and in only one patient without stroke. Recently, we also described a 43‐year‐old man who developed cerebral infarction accompanied by both hypereosinophilic syndrome (HES) and PFO. The source of emboli in this patient was determined and HES‐associated embolic events were precluded via laboratory and radiological examination. A OCT‐documented thrombus lodged in the PFO, incriminating the stroke in this patient to PFO but not HES.^27^ Microthrombi within the PFO might be caused by a slow flow and hypercoagulability in the foramen ovale, leading to thrombus formation in situ (Figure 1), which may constitute a new mechanism underlying PFO‐mediated paradoxical embolism. Therefore, the application of OCT can clearly show the microstructure of PFO, expanding our understanding of pathophysiological mechanisms underlying paradoxical embolism. However, OCT is not mandatory in all PFO patients and should not be used as a basis for determining whether to occlude a PFO.

Increasing evidence has shown an association between PFO and cerebral events, including stroke, transient ischemic attack (TIA), and headache. Although most paradoxical emboli travel to the brain, noncerebral paradoxical systemic embolic events are also associated with PFO, which accounts for approximately 5−10% of all paradoxical embolisms.^28^ Paradoxical emboli can enter the coronary, visceral, or peripheral (upper and lower extremities) circulation through a PFO, causing acute myocardial infarction (AMI), renal infarction, and peripheral ischemia. In one retrospective cohort study conducted from 2001 to 2009, 416 patients with PFO were included, in whom 219 patients (52.6%) presented with cryptogenic stroke, 38 patients (9.1%) with migraine headaches, and 80 patients (19.2%) with transient neurological deficits consistent with TIA or complex headache.^29^ In addition, 12 patients (2.9%) developed noncerebral embolism, including eight cases with AMI without angiographic evidence of obstructive coronary disease assessed and four with peripheral arterial or retinal arterial embolism.^29^ These results indicated that PFO‐mediated paradoxical embolism is a potential cause of multiorgan or tissue infarctions. As paradoxical embolism can lead to adverse outcomes in many organs or tissues in patients with PFO, early diagnosis and treatment of PFO are required to prevent catastrophic events. However, a well‐accepted criteria for the diagnosis of paradoxical embolism are still lacking.

#### 2.2.1 PFO and stroke

**FIGURE 2:**
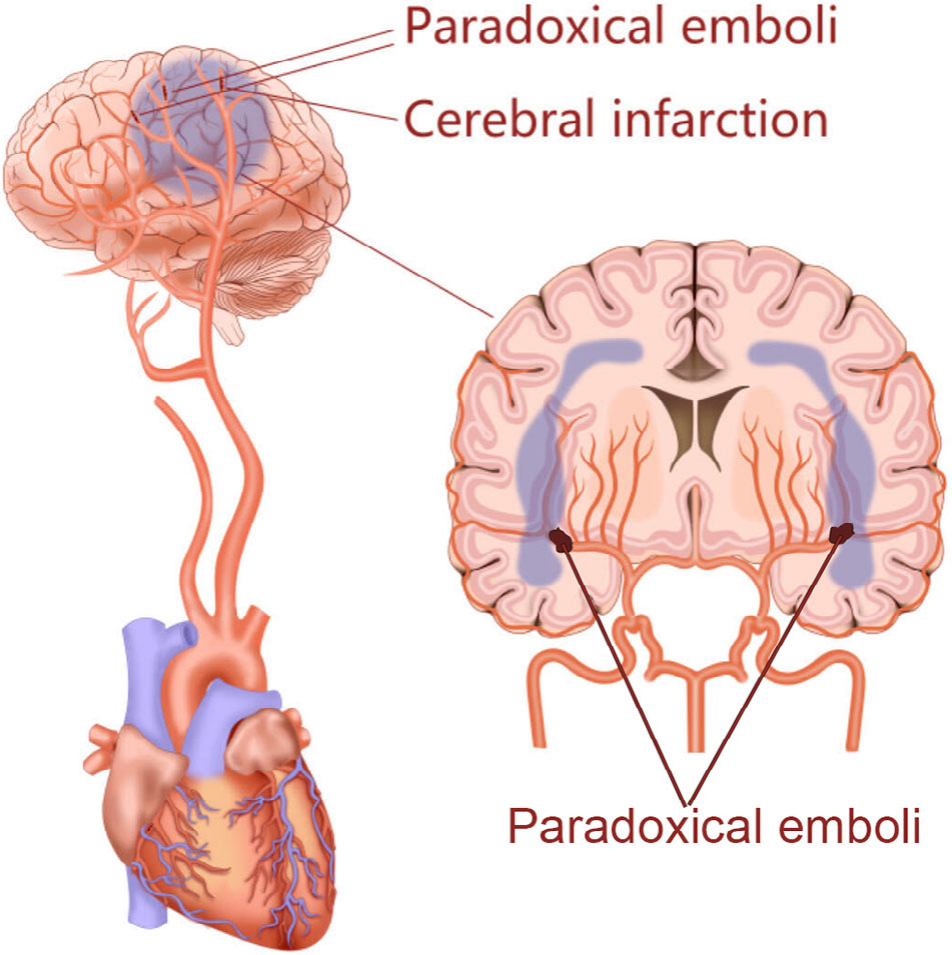
Patent foramen ovale (PFO)‐mediated ischemic stroke. An embolus originating from the vein travels through PFO to the cerebral arteries causing ischemic stroke.

The brain receives 15% of the cardiac output and is extremely sensitive to ischemia.^30^ Stroke is a one of the most frequent causes of mortality and long‐term disability worldwide. Each year in the United States, an estimated 700,000 inhabitants experience stroke, with 500,000 events being the first attack and 200,000 events recurrent one.^31^ In addition, it is estimated that the incidence of TIA ranges from 68.2 to 83 per 100,000 people and about 15% patients with strokes are heralded by TIA.^31^ Most stroke are of ischemic origin, in which atherosclerosis, atrial fibrillation, and arteritis are the main causes. However, in 26−40% of ischemic strokes and in 64% of patients less than 55 years, no explanatory reasons were found, which were named cryptogenic stroke.^32^ Cryptogenic stroke usually occurs in younger population with few identifiable etiologies such as atherosclerosis and atrial fibrillation, but recent studies have revealed that paradoxical embolism involve in the pathogenesis of cryptogenic stroke. When the foramen ovale is patent, the thrombus from the vein system may travel through the “hole” into the cerebral arteries and cause stroke (Figure 2). Patients with cryptogenic stroke are always younger, and have a higher incidence of PFO compared with these patients with stroke of definite origin.^33^ Accumulating evidence indicates that PFO can be found in almost 50% of cases with cryptogenic stroke, and has become an independent risk factor for cryptogenic stroke.^32^, ^34^, ^35^ Considering that cryptogenic stroke accounts for about one quarter of all ischemic strokes, PFO may cause about 5% of all ischemic strokes and 10% of all ischemic strokes among young patients (≤55 years).^36^ Recently, evidences indicated that PFO was independently associated with cryptogenic stroke not only in younger patients (<55 years) but also in older patients (≥55 years).^37^ In one study involving 503 patients with stroke, the prevalence of PFO was significantly greater in older patients (≥55 years of age) with cryptogenic stroke than those with stroke of definite origin (28.3 vs. 11.9%) or than younger patients (43.9 vs. 14.3%).^37^ Thus, both young and older patients with cryptogenic stroke should be screened for PFO.

Although an association between PFO and stroke has been repeatedly demonstrated by cross sectional studies, such a relation has been substantiated by few longitudinal studies. The Olmsted County Study enrolled 585 randomly selected individuals who were over 45 years of age, and PFO was identified in 140 adults (24.3%). Over 5.1 years of median follow‐up, PFO was denied as an independent predictor of future cerebrovascular events (including cerebrovascular disease‐related death, ischemic stroke, and TIA) in the general population after correcting for age and comorbidities.^38^ However, among the 1100 stroke‐free population aged 39 years or old in the Northern Manhattan Study, only 164 (16.9%) individuals had PFO and no significantly association was observed between PFO and the occurrence of stroke after a follow‐up of 79.7 ± 28.0 months on average.^39^ A selection bias in the enrolled population may have resulted in the observed discrepancies.

The size of the PFO may be a key modifying factor for stroke risk, and a large PFO may confer a higher risk of stroke than a small PFO. In an early study by Steiner et al.,^40^ the incidence of medium and large PFO was higher in patients with cryptogenic stroke than in those with identifiable causes of stroke (26 vs. 6%). Additionally, they found that patients with stroke and a large PFO exhibited more cerebral imaging features of embolic infarcts than those with a small PFO, including more superficial infarcts (50 vs. 21%), larger infarcts (>1 lobe, 14 vs. 2%), more occipital and infratentorial strokes (57 vs. 27%), and more posterior circulation involvement (64 vs. 33%), suggesting that a larger PFO may be more likely to cause paradoxical embolization.^40^ However, conflicting results were published in later studies. The Risk of Paradoxical Embolism study did not show a relationship between PFO size and PFO‐attributable stroke, suggesting that TEE alone has some limitations in the risk stratification of PFO based on anatomic features.^41^

Epidemiological studies found that the patients with stroke remained at a high risk of recurrent ischemic stroke.^35^, ^42^ In a meta‐analysis of 48 observational comparative studies, individuals with cryptogenic stroke or TIA and concomitant PFO who received medical therapy alone had a higher incidence of recurrent neurological events.^42^ Thus, identify those patients at a higher risk of recurrent stroke is important. Among patients with PFO, the presence of atrial septal aneurysm (ASA), anatomical degree of patency, and magnitude of microbubble passage have been shown to increase thromboembolic risk and are related to an enhanced risk of recurrent stroke.^30^, ^36^ Most patients with PFO have an ASA and the annual risk of recurrent ischemic stroke in patients with ASA is about 2.5% annually.^43^ In one study, 98 patients who underwent a previous stroke were enrolled and 65 (66.3%) patients had moderate‐to‐severe ASA and basal shunts. Logistic regression analysis showed that moderate‐to‐severe ASA score was the most powerful predictor of left atrial dysfunction in patients with PFO, which contributed to embolization in these patients.^44^ In addition, ASA may result in a more frequent and wider opening of the PFO tunnel, which promotes a R‐L shunt by redirecting flow from the inferior vena cava to the PFO, contributes to thrombus formation on the surface of the ASA and facilitates paradoxical embolus passing through the PFO.^43^, ^45^ Therefore, patients with ASA have a higher risk of paradoxical embolism compared with patients without ASA. Recently, Turc et al.^43^ enrolled 898 participant with PFO and stroke and confirmed that the ASA is an important predictor of recurrent stroke in patients with recent PFO‐mediated ischemic stroke. However, they also failed to document an independent relation between time to stroke recurrence and PFO size, and there was no evidence of a synergistic effect of ASA and a large PFO.^43^

PFO‐mediated stroke is primarily considered to be a consequence of a paradoxical embolism originating from a DVT.^46^ The thrombus “in transit” across the PFO can result a cerebral vascular event, including cryptogenic stroke, TIA, and migraine headaches. It has been shown that a 1 mm thrombus is sufficient to cause the occurrence of stroke.^36^ In the PELVIS study, 95 patients with stroke underwent magnetic resonance imaging (MRI) examination within 72 h of the onset of new symptoms, and an increased prevalence of pelvic DVT was found in patients with cryptogenic stroke relative to those with stroke of definite origin, suggesting that in some patients, cryptogenic stroke might be caused by paradoxical thromboembolism from the pelvic veins via the PFO.^33^ Moreover, studies have indicated that a history of DVT or pulmonary embolism, recent prolonged travel, Valsalva maneuver preceding the onset of focal neurological symptoms, and waking up with stroke or TIA were independently associated with PFO‐mediated cerebrovascular events.^47^ However, the mechanisms remain unclear, and it is difficult to assess whether a PFO is incidentally or causally associated with stroke in a given case.^47^, ^48^ When a patient has coexistent PFO and cryptogenic stroke, clinical evidence of silent thromboembolism should be carefully investigated.

In addition, a few cases of accidentally reported paradoxical air embolism through a PFO have been reported. Cerebral air embolism after central venous catheter placement is a rare but hazardous complication, where air bubbles pass from the veins to the arterial system via a PFO. The sequelae were mainly dependent on the amount and location of embolized air. A 54‐year‐old woman died suddenly after the removal of a jugular venous catheter by herself, and multiple gas emboli in the pulmonary, coronary, and cerebral arteries were found on postmortem computerized tomography (CT) scanning, suggesting the occurrence of acute ischemia of the heart and brain caused by massive air inflow to the arterial circulation through a PFO.^49^

Taken together, as paradoxical embolism in patients with PFO is a potential mechanism of stroke, the role of PFO should be carefully evaluated in patients with cryptogenic stroke. However, it is poorly understood why an overwhelming majority of paradoxical embolisms are related to PFO and present as strokes, since blood flow to the cerebral vessel accounts for only 15% of cardiac output. Further research is required to clarify this issue.

#### 2.2.2 PFO and myocardial infarction

**FIGURE 3:**
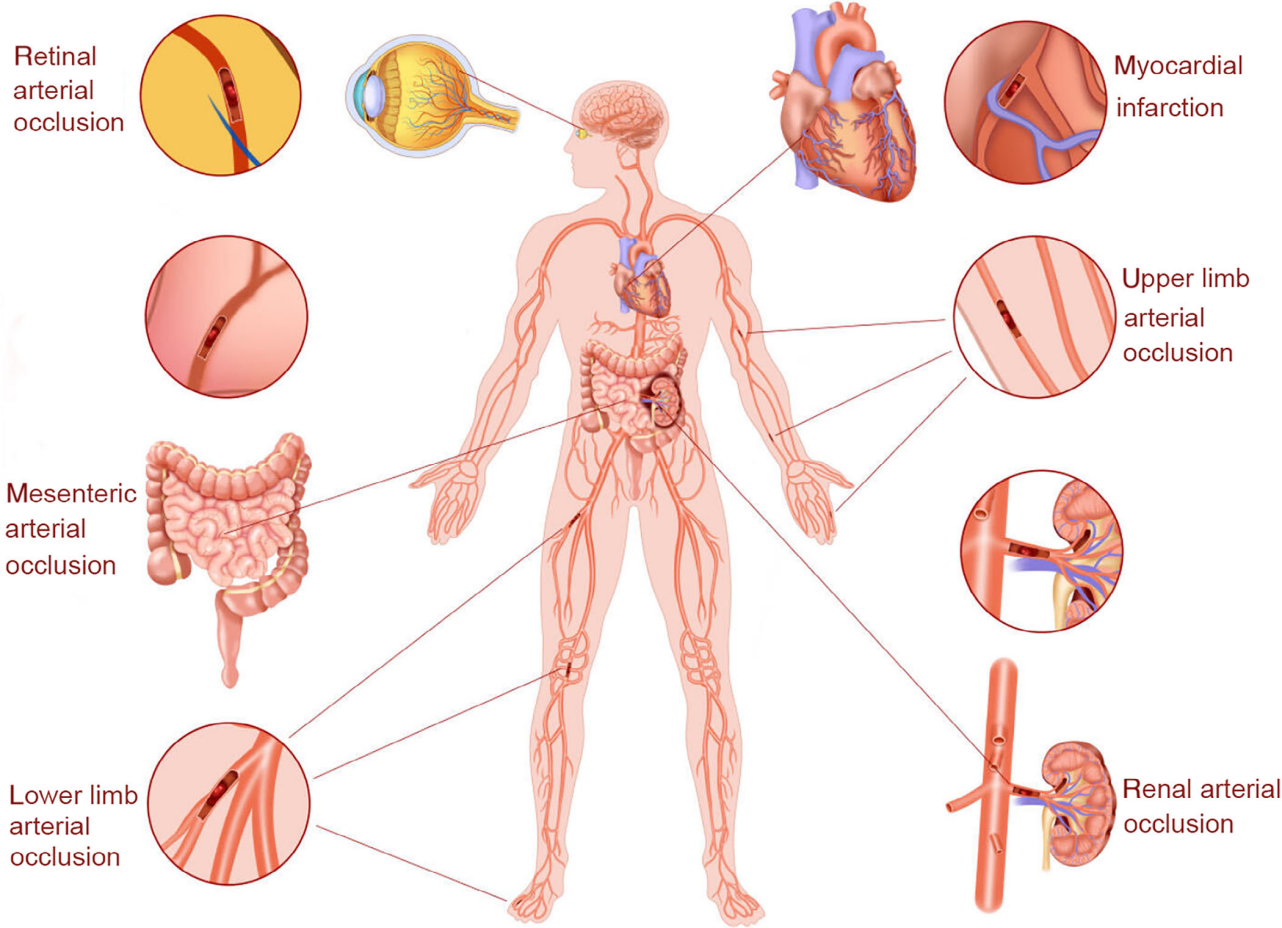
Clinical manifestations of patent foramen ovale (PFO)‐mediated paradoxical embolism besides stroke. Emboli from the vein enter the arterial circulation through PFO causing acute myocardial infarction, and occlusion of the retinal, renal, peripheral, and mesenteric arteries.

The prevalence of paradoxical embolism varies with different organs involved (Figure 3). In general, paradoxical embolism involves mainly the brain and much less the coronary, retinal, and renal arteries.^50^ Coronary embolism is an uncommon cause of occlusive coronary artery disease (CAD), and the embolic source mainly originates from the heart. However, coronary emboli may also come from PFO, leading to a complete or near‐complete occlusion of the major coronary artery. It has been reported that among all cases of paradoxical embolism, coronary embolism accounts for 10−15%.^51^ Thus, immediately establish a diagnosis and identify the source of the emboli in suspected patients is especially important.

In one retrospective study, among 426 patients with PFO, eight (1.9%) presented with AMI without evidence of obstructive coronary disease as assessed by angiography.^29^ Shah et al.^52^ systematically analyzed 386 patients with a thrombus straddling a PFO and found that 18 patients (4.6%) had AMI, in whom the left anterior descending artery (LAD) was the most frequently affected artery and most emboli were located in the proximal LAD. Kleber et al.^53^ performed a large retrospective study and screened a hospital database of 4848 patients with AMI over 10 years. They found that only 22 (0.45%) patients had a presumed paradoxical coronary embolism due to PFO. In a larger study that included 1651 patients with AMI, 11 (0.67%) were presumed to have paradoxical coronary embolism, and <1% AMI was associated with PFO‐mediated paradoxical embolism.^53^ The different incidence of paradoxical coronary embolism secondary to PFO may be due to heterogeneous populations with different inclusion criteria. Considering that there are approximately 7 million AMI cases worldwide annually, it is estimated that at least 35,000 cases were caused by paradoxical coronary embolism.^53^ Although the percentage was significantly low, paradoxical embolism should not be ignored as a cause of AMI, because the prevention and treatment strategies in these patients are fundamentally different from those in patients with AMI due to atherosclerosis.^53^

In the literature, PFO‐mediated coronary paradoxical embolism as the cause of AMI is mainly limited to case reports, and a systematic summary was absent. In this review, we systemically assessed the association between AMI and PFO‐mediated coronary paradoxical embolism. We found that most patients with paradoxical coronary embolism presented with ST‐elevation myocardial infarction rather than non‐ST‐elevation myocardial infarction, and the infarct territory involved mainly inferior or posterior left ventricular wall.^53^ While previous studies revealed that most coronary emboli lodged in the LAD artery,^54^ we found that in most cases of presumed PFO‐mediated coronary paradoxical embolism, the emboli were located in a distal or branch segment of the right coronary artery (RCA), followed by the left circumflex artery (LCX), and least in the LAD.^55^, ^56^, ^57^ For example, Ghafoor et al.^55^ described a 33‐year‐old woman with acute inferior myocardial infarction presenting with occlusion of the distal RCA on coronary angiography due to coronary paradoxical embolism secondary to PFO. In another study by Wilson et al.,^58^ a 28‐year‐old man with AMI and coronary paradoxical embolism in the branch of the LCX secondary to PFO was reported. However, few studies have reported embolism of LAD or left main coronary artery. Surprisingly, Meier‐Ewert et al.^59^ reported a 62‐year‐old woman with near‐complete occlusion of the left main coronary artery, a totally occluded LAD, and a partially occluded LCX due to paradoxical embolism through a PFO.

Although many cases have been published, it is difficult to obtain direct evidence of paradoxical embolization in the coronary arteries, such as a mobile thrombus crossing a PFO. Cardiac magnetic resonance imaging (CMRI) is a noninvasive technique for detecting focal infarctions in the coronary artery territories. Liang et al.^60^ reported a case of a 44‐year‐old man who presented with AMI but nonobstructive coronary arteries on angiography. However, CMRI revealed multiple focal infarctions in the RCA and LCX arteries. Based on the combination of multiterritorial infarction, nonobstructive coronary arteries, and PFO, a paradoxical embolism was suspected.^60^

In addition to direct embolism in the coronary arteries, other factors may also contribute to the pathogenesis of PFO‐mediated AMI. Dao and Tobis^29^. suggested that PFO‐mediated AMI in some cases was probably caused by vigorous coronary vasospasm, not by a paradoxical embolus. They thought that a vasoactive substance bypassed lung metabolism in the lungs and reached the arterial circulation through a PFO, inducing intense coronary spasm and AMI.^29^

Taken together, the incidence of paradoxical coronary embolism appears low, and the diagnosis of PFO‐mediated coronary embolism remains a challenge because the diagnostic criteria in this setting are yet to be established. However, considering a large number of patients with AMI worldwide, paradoxical coronary embolism as a possible cause of AMI should not be ignored in the presence of PFO. On the contrary, paradoxical coronary embolism should be highly suspected, particularly in patients presenting with AMI and a high thrombus burden without evidence of coronary atherosclerosis in the setting of a PFO.

#### 2.2.3 PFO and retinal arterial occlusion

Retinal artery occlusion (RAO) is an emergent ophthalmic disease that can cause sudden vision loss in the affected eye. RAO mainly occurs in older adults rather than in young and healthy adults.^61^ As an uncommon vaso‐occlusive disorder, RAO shares pathophysiology with acute ischemic stroke, which is mainly caused by atherosclerosis, thromboembolism, and arteriospasm.^62^ For physicians, the cause of RAO should be quickly determined, as timely evaluation and intervention are important to avoid permanent vision loss.

Paradoxical thromboembolism through the PFO as a possible reason for RAO has been reported in several studies, which mainly occurred in relatively younger individuals. A clot passed from the peripheral veins to the left heart and arterial circulation via the PFO and eventually blocked the retinal artery of the affected eye, resulting in RAO, which includes branch RAO (BRAO) and central RAO (CRAO).^63^ In 2013, Shoeibi et al.^64^ described a 29‐year‐old female who presented with a sudden superior visual field defect in the left eye and was diagnosed with BRAO on the basis of an ophthalmologic examination. Further transthoracic echocardiography (TTE) examination revealed a small PFO, which was supposed to have resulted in paradoxical embolism and BRAO. This is the first report on the association between BRAO and PFO.^64^ Subsequently, Iqbal et al.^61^ showed that a 31‐year‐old man acutely developed left‐sided decreased and blurred vision due to BRAO resulting from paradoxical embolism and concomitant PFO. After administration of antiplatelet therapy, his vision progressively improved during hospitalization, and the patient recovered well during follow‐up.^61^ Recently, Si and Wang^65^ from our hospital reported an interesting 40‐year‐old female case presenting multiple RAOs following injection of hyaluronic acid as facial filler into the nasal root, which confirmed by a series of ophthalmologic examinations, and a PFO was determined by TEE. Fortunately, the patient's visual acuity improved quickly within 3 days after aggressive treatment.^65^

Compared with BRAO, CRAO may lead to a more severe outcome because the inner two‐thirds of the retina lose their blood supply.^66^ CRAO always presents as acute and permanent vision loss in one eye. Importantly, inner retinal infarction occurs within 12−15 min, if total central RAO.^67^ Once CRAO lasts for approximately 4 h, massive and irreversible retinal damage, including optic nerve atrophy, nerve fiber damage, and vision loss will occur.^67^ In 2021, a 43‐year‐old man with sudden painless right‐sided vision loss was reported, who was diagnosed with CRAO. After a PFO was verified by TTE examination, they believed that a silent venous thromboembolism traveling through a PFO accounted for the catastrophic event.^68^ Finally, the PFO was successfully closed without any complications. Sabanis et al.^66^ also reported that a 62‐year‐old male patient without a previous medical history of eye and nerve diseases presented with left retinal whitening and macular edema compatible with an acute CRAO secondary to PFO due to paradoxical embolism.

As there exist extensive reports on the correlation between RAO and PFO, PFO should be considered in younger or healthy patients with RAO without other etiological risk factors. The immediate screening and diagnosis of PFO in these patients can help decrease the risk of future ocular or systemic embolic events and associated morbidities. However, the casual relationship between PFO and RAO remains to be further established.

#### 2.2.4 PFO and renal arterial occlusion

Most acute renal infarctions are caused from the acute occlusion of the renal arteries, where paradoxical embolism is a rare etiology. However, several reported cases have shown that venous thrombi can pass through a PFO and occlude the renal artery. In 1999, a 67‐year‐old woman presented with bilateral main renal artery occlusions and renal infarctions was reported, in whom continuous opening of the PFO presumably permitted deep vein thrombi to arrive to the systemic and renal artery.^69^ Later, Iwasaki reported that a 60‐year‐old man experienced sudden onset of right flank pain during golf practice, who had no medical history of atherosclerotic diseases, and PFO was detected using TTE. Paradoxical embolism caused by a venous thrombus passing through a PFO was responsible for cryptogenic renal infarction.^70^ Recently, Lim et al.^71^ described a 62‐year‐old man with bilateral renal artery thromboembolim due to a PFO‐associated paradoxical embolism. Notably, these cases exhibited that paradoxical renal embolism was usually bilateral.^69^ Interestingly, PFO‐mediated paradoxical embolism may also occur in patients undergoing renal transplantation. A 56‐year‐old man underwent live renal transplantation, and 4 weeks later, the patient developed acute renal allograft dysfunction with elevated serum creatinine levels. TTE revealed the presence of a PFO with a spontaneous R‐L shunt without ventricular thrombi. Consequently, a paradoxical embolism was deemed responsible for renal allograft failure. After PFO closure and anticoagulation therapy, the patient presented with normal renal perfusion and decreased serum creatinine levels during follow‐up, consistent with a previous renal infarction.^72^

Despite these aforementioned reports, the major source of the renal artery embolism was not proven, and the diagnoses were almost presumptive. However, in cases in whom the occurrence of acute renal infarction is impossible due to thrombosis or traditional embolism, a potential PFO‐mediated paradoxical embolism should be suspected. Unfortunately, the association between PFO‐mediated paradoxical embolism and renal infarction has been limited to individual case reports, and no case‐control study has yet been conducted. Therefore, future research should focus on this association.

#### 2.2.5 PFO and peripheral arterial occlusion

Atherosclerosis remains the predominant cause of arterial disease of the extremities, and limb ischemia induced by embolic occlusion is rare. However, critical limb ischemia caused by a thrombus can result in severe morbidity and mortality, which may require urgent embolectomy. A PFO provides an anatomical substrate for a thrombi that develops in the systemic venous circulation arrive into systemic circulation. Depending on the embolization site, paradoxical emboli can cause acute ischemia of the limbs, albeit often minor. Thus, a paradoxical embolus via a PFO has been regarded as an important cause of acute limb ischemia.

Most cases of acute limb ischemia occur in the lower limbs. Previous studies reported that 72% of patients with PFO‐mediated paradoxical embolism developed lower limb ischemia.^73^ In 2019, a 67‐year‐old man complained of acute left leg pain and numbness, and thrombotic occlusion of the left superficial femoral artery at the distal end was confirmed by angiography. TTE revealed the presence of a PFO, which was responsible for the paradoxical embolism in acute lower limb ischemia, and no embolic events recurred after percutaneous closure of the PFO.^74^ Chughtai et al.^75^ first revealed a case of a 27‐year‐old African American man with a postoperative paradoxical embolism. The patient had bilateral pulmonary artery emboli and underwent surgical embolectomy. Postoperatively, the patient developed acute left lower extremity ischemia due to embolic arterial occlusion, and a thrombus on the mitral valve and a PFO were observed on TEE, suggesting a paradoxical embolism through the PFO that accounted for this thromboembolic event.^75^ Therefore, PFO‐mediated paradoxical embolism may be evaluated in unexplained cases of acute limb ischemia.

Acute upper limb ischemia is relatively uncommon, accounting for only one‐fifth of all acute ischemia of the extremity.^76^ Kallel et al.^76^ reported a female patient aged 69 years manifesting with acute upper limb ischemia with thrombosis of the left humeral artery extending to the radial and ulnar arteries, as assessed by CT angiography, which was secondary to DVT and pulmonary embolism in the presence of PFO.^76^ Shahi and Nair^77^ also reported an uncommon case of ischemic fingers due to thrombus occlusion in the digital vessels of the right index and middle fingers in a young woman, which was ascribed to lower‐limb DVT associated with a paradoxical embolus through a PFO.

Occurrence of two paradoxical embolic events in the lower and upper limbs secondary to PFO were also present.^78^ However, multiple limbs were affected in only a minority of cases. Additionally, paradoxical thromboembolic events may occur in cases of organ transplants. Recently, Haghikia et al.^72^ presented a man aged 56 years with renal transplantation who developed embolic occlusion in both the left common femoral artery and right popliteal artery caused by PFO‐associated paradoxical embolism.

After atherosclerotic risk factors are excluded, patients with acute limb ischemia should be evaluated for embolic etiology. Paradoxical embolism should be born in mind when the embolus source is not evident, particularly in young and middle‐aged individuals. Although PFO is always evaluated in patients with cryptogenic stroke, PFO‐mediated paradoxical embolism is often ignored as a cause of limb ischemic events. Thus, clinicians should keep PFO in mind as a potential reason in otherwise unexplained cases of acute limb ischemia.

#### 2.2.6 PFO and arterial occlusion in other organs

Paradoxical embolism through PFO can also affect arteries of other organs, such as splenic and mesenteric arteries. A 61‐year‐old male patient with bilateral pulmonary embolism presented with multiple splenic infarcts and right renal ischemia, as assessed by CT angiography.^79^ TEE and TTE revealed a mobile thrombus entrapped in the PFO, which was responsible for the thromboembolic event.^79^

The association between mesenteric artery occlusion and PFO‐mediated paradoxical embolism has seldom been reported. Critical mesenteric ischemia caused by paradoxical embolism is an emergency that requires embolectomy. A 76‐year‐old man with DVT in the left inferior limb complicated by bilateral pulmonary embolism was reported in 2021. Abdominal angiography revealed thrombus occlusion in the superior mesenteric artery. They believed that a suspected paradoxical embolism due to PFO accounted for the acute superior mesenteric and right upper limb ischemia.^80^

#### 2.2.7 PFO and multiple organ embolism

Theoretically, paradoxical emboli passing through the PFO may embolize multiple organs simultaneously, although these events are rare. For example, emboli can enter the cerebral and coronary arteries simultaneously, inducing cerebral and myocardial ischemia.^81^ Recently, a 47‐year‐old female patient who presented with transient motor dysphagia and worsening chest pain with ST elevation in the inferior leads of electrocardiogram was presented. The women underwent urgent coronary angiography, which showed the LCX was occluded by thrombus. Subsequently, brain MRI revealed hyperintense regions in the left cortex. In this patient, paradoxical emboli through the PFO were suspected after a PFO and a deep venous thrombus in the left leg were found.^82^ In addition, small myocardial infarction may be subclinical and patients with this pathology may be asymptomatic. In a previous study, 74 patients with PFO and a first cryptogenic cerebral ischemic event without a clinical history of myocardial infarction were enrolled and underwent CMRI, which showed that eight (10.8%) patients had subclinical myocardial infarction accompanying cryptogenic stroke.^81^

Simultaneous involvement of three or more organs has also been reported. In one study by Islam et al.,^50^ a 62‐year‐old woman with acute pulmonary embolism presented with an acute cerebral infarct, left axillary and brachial artery emboli, and inferior wall myocardial infarction caused by a paradoxical embolism through the PFO.

### 2.3 Diagnosis of PFO

Right heart catheterization allows a guidewire to cross the PFO, which has been considered the most accurate technique for determining the presence of a PFO.^35^ However, this procedure is invasive and cumbersome. Transcranial Doppler (TCD), TTE, and TEE applied with microbubble contrast are noninvasive techniques for PFO detection. Among these, TCD is a safe and reproducible technique for predicting a PFO. Aerated saline was injected into the peripheral vein of the subject, and microbubble signals were detected on Doppler assessment of the brain‐supplying artery in the presence of a R‐L shunt.^17^ Previous studies reported that the sensitivity and specificity of TCD for the detection of PFO were 97 and 93%, respectively.^83^ However, TCD cannot distinguish an intracardiac from an intrapulmonary shunt, nor evaluate the morphology of a PFO.^83^

The use of TTE is widespread because of its noninvasiveness and high availability. Ample evidence showed that the sensitivity and specificity of TTE for determining PFO were 46 and 99%, respectively, suggesting a low sensitivity and a high specificity for this modality.^84^ TEE can directly display PFO morphology, including anatomic size and tunnel length, and is superior to TTE in identifying PFO, with nearly a 100% sensitivity and a 100% specificity.^17^, ^83^ Moreover, blood shunting sometimes can be directly discovered using color Doppler. However, a recent meta‐analysis showed that TCD is more sensitive than TTE for detecting PFO in individuals with cryptogenic cerebral ischemia.^85^

Contrast TTE and contrast TEE can assess the degree of R‐L shunt with intravenous administration of aerated saline based on the presence of microbubbles in the left heart after right atrial opacification.^25^ These evaluations can be conducted at rest and during Valsalva maneuver, which serves to raise right atrial pressure and promotes a R‐L shunt across PFO. In normal subjects without PFO, microbubbles are filtered by the lungs and are not observed in the left atrium. Thus, contrast studies have been commonly incorporated into routine echocardiographic evaluation of PFO. Now, improved echocardiography and TCD technologies significantly increased the diagnostic accuracy.

### 2.4 Treatment of PFO

Currently, medical therapies, including anticoagulation and antiplatelet therapy, and surgical and percutaneous transcatheter PFO closure, are the main therapeutic strategies for patients with stroke and PFO. Although paradoxical embolization involved in the pathogenesis of stroke combined with PFO, oral anticoagulation did not show convincing superiority over antiplatelet therapy. In 2002, data from the PICSS indicated that therapy with warfarin or aspirin resulted in similar rate of recurrent stroke or death in stroke patients with PFO.^86^ Recently, a meta‐analysis that included five randomized controlled trials and 1720 patients with stroke or TIA of undetermined cause and PFO showed that no apparent discrepancy was observed in stroke recurrence between anticoagulant‐assigned and antiplatelet‐assigned patients (1.73 vs. 2.39 per 100 patient‐years).^87^ Considering the increased risk of bleeding from warfarin compared with aspirin, antiplatelet therapy may be favored in most patients with stroke and PFO.^17^ Although novel oral anticoagulants display a lower bleeding risk than warfarin, they provide no clear superiority than aspirin in preventing the occurrence of recurrent stroke in patients with embolic stroke of an undetermined source and PFO.^88^ Long‐term antithrombotic therapy, including both anticoagulation and antiplatelet therapies, increases the risk of bleeding.

Transcatheter techniques to close the PFO are safe and effective procedures for the secondary prevention of paradoxical embolism, which are related to a lower incidence of complications and further embolic events.^89^ In recent years, a dramatic surge in transcatheter PFO closure has been performed. However, whether PFO should be closed in patients with stroke remains controversial. In 2012−2013, three multicenter randomized clinical trials, including CLOSURE, RESPECT, and PC, were conducted in patients with cryptogenic stroke or TIA who had a PFO.^90^, ^91^, ^92^ Unfortunately, PFO closure did not present any superiority than medical therapy for secondary prevention of stroke or TIA in patients with PFO in these trials. On the other hand, a meta‐analysis reported that transcatheter closure of the PFO remarkably decreased 41% of recurrent neurological events (including stroke, TIA, or both) compared with medical therapy. However, PFO closure did not display clear clinical advantage when assessing stroke alone, suggesting a nonsuperior effect of PFO closure against recurrent stroke.^93^ Recently, the CLOSE study revealed that PFO closure resulted in a lower rate of stroke recurrence in patients with stroke attributable to PFO characterized by an ASA or large interatrial shunt than in patients receiving medical therapy alone.^94^ Subsequently, the Gore REDUCE Clinical study and RESPECT study also showed that percutaneous closure of PFO significantly decreased the incidence of recurrent ischemic stroke among younger individuals (<60 years) with a cryptogenic stroke than antiplatelet therapy during extended follow‐up.^95^, ^96^ These trials suggested that medical therapy alone did not reduce stroke recurrence over time, and PFO closure is recommended for patients aged less than 60 years with cryptogenic stroke and a large PFO or an obvious R‐L shunt.^97^ The different enrolled subjects, short follow‐up periods, and low recurrent ischemic events may explain the nonsuperiority of PFO closure over medical therapy in the CLOSURE, RESPECT, and PC trials.^98^ Based on recently published data including three major trials, a meta‐analysis that enrolled 3560 patients by Turc et al.^99^ indicated that PFO closure was associated with a 64% lower risk of stroke recurrence than medical therapy, and thus superior to antithrombotic therapy in protecting against the recurrence of stroke in patients aged up to 60 years with cryptogenic stroke. Unfortunately, an elevated risk of atrial fibrillation was observed after PFO closure.^99^

However, the use of PFO closure in older patients with cryptogenic cerebral embolism is still controversial. In one retrospective study, 335 patients with paradoxical embolism who received PFO closure were enrolled, including 120 (36%) patients aged >55 years and 215 (64%) younger patients. During a median follow up of 4 years, both recurrent stroke and TIA or stroke alone was significantly lower in younger patients than in older patients (>55 years), suggesting PFO closure was more effective in preventing recurrent stroke and TIA in young than in older patients.^100^ In addition, univariable analysis showed that age >55 years, the presence of atherosclerotic risk factors (≥2), and multiple paradoxical embolic events prior to PFO closure predicted recurrent stroke or TIA. Importantly, multivariate analysis indicated that age >55 years remained an independent predictor of recurrent stroke or TIA. Thus, older age promotes the recurrence of thromboembolic events, and PFO closure may be less beneficial in elderly population.^100^ However, different conclusions were drawn in later studies. Wintzer‐Wehekind et al.^101^ followed 475 patients with cryptogenic embolism for a mean of 8 years to evaluate the efficacy and long‐term safety of PFO closure in adults above 60 years. The results showed that transcatheter closure of PFO is safe and result a decreased rate of ischemic events in older patients (>60 years).^101^ Another multicenter study included 388 (>60 years) and 883 (≤60 years) patients with cryptogenic ischemic event who underwent a PFO closure. After a follow‐up of 3 years on average, a relatively low rate of recurrent ischemic events was observed after PFO closure in patients >60 years, compared with those patients who did not receive PFO closure.^102^ Therefore, PFO closure may not be excluded in older patients, even though atherosclerotic risk factors may be more frequent in this age group. Considering the heterogeneity of the above results, multicenter randomized studies for evaluating the efficacy of PFO closure in elderly patients are warranted.

The etiology and pathogenesis of stroke are multifactorial, and confirming that PFO is an established cause of the event is difficult, especially if other possibilities have not been fully excluded. Nonetheless, some randomized and multicenter studies have established a consistent pattern in favor of PFO closure in preventing stroke recurrence compared with medical therapy, particularly in younger patients. However, even in patients with a cryptogenic stroke, PFO may be an incidental finding without a causal relationship, and consequently, PFO closure may not be beneficial in these unrelated subjects.^21^, ^103^ Moreover, the risk of stroke recurrence in patients with PFO is considerably lower than that in patients with other common stroke mechanisms. Thus, determination of stroke‐related PFO is particularly important, and clinicians should exclude alternative mechanisms of stroke when considering PFO closure. In addition, it remains unclear whether stroke patients aged > 60 years or those with combined atherosclerosis can benefit from PFO closure. Therefore, determining whether PFO closure should be offered to older patients with PFO and cryptogenic stroke is particularly challenging. Finally, the diagnosis of noncerebral paradoxical embolism is often presumed, and whether PFO closure is beneficial for decreasing the recurrence of extracerebral embolic events in patients with noncerebral embolic manifestations remains unclear. Future clinical studies should address these questions.

## 3 ATRIAL SEPTAL DEFECT

### 3.1 Pathophysiology of ASD

#### 3.1.1 Epidemiology, anatomy, and classification of ASD

As a congenital defect of the atrial septum, ASD is caused by abnormal development during the embryonic period and accounts for approximately 10−15% of individuals with CHD.^104^ This congenital disorder is always asymptomatic until adulthood and may be identified incidentally. A substantial proportion of patients with ASD can exhibit some nonspecific manifestations, including shortness of breath, dyspnea on exertion, exercise intolerance, or palpitations, especially when the defect is larger.^105^ A small proportion of subjects with ASD have systemic emboli, which may lead to stroke, due to paradoxical embolization. In patients with ASD, a left‐to‐right (L‐R) shunt is formed across the atrial septum due to the higher pressure in the left atrium.^106^ The degree of the shunt is mainly determined by the size of the ASD, compliance of both ventricles, and pressure of both atria. Although small ASDs may be hemodynamically insignificant and do not cause dilation of the right heart structures, larger and long‐standing defects can lead to hemodynamic abnormalities if left untreated. Significant shunts trigger a cascade of pathological alterations in cardiac chambers and pulmonary vasculature, including an increased in the right atrial pressure, right‐sided enlargement, and pulmonary hyperperfusion, which may lead to damage to the right atrial reservoir, pulmonary vascular remodeling, and elevated pulmonary vascular resistance (PVR), ultimately resulting in heart failure and pulmonary arterial hypertension (PAH) in larger and untreated ASD.^107^, ^108^ Compared with general community, patients with ASD have a higher overall mortality due to congenital malformations, stroke, heart disease, other diseases of the circulatory, and disease of the endocrine and respiratory systems, even after ASD closure.^109^

**FIGURE 4:**
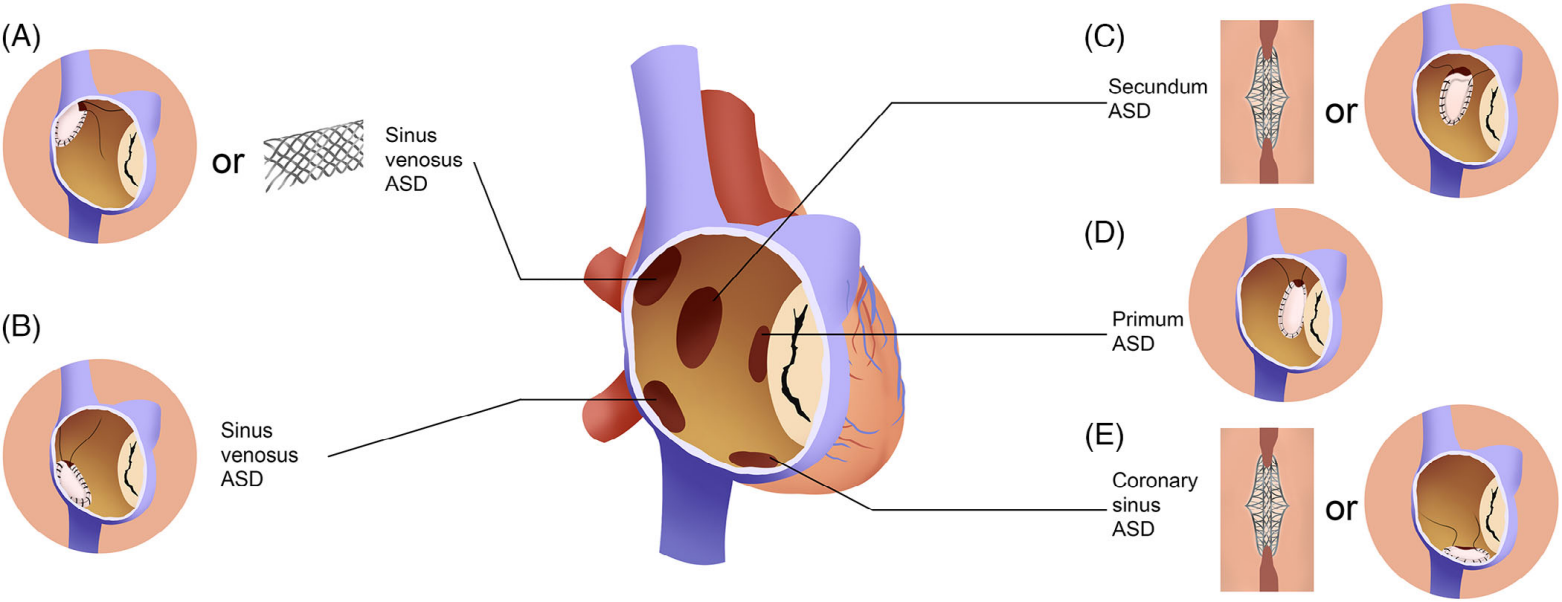
Pathological type and treatment options of atrial septal defects (ASDs). Types of ASDs include ostium secundum (A), ostium primum (B), sinus venosus (C), and coronary sinus (D) defects. Treatment strategies are based on ASD type and morphology.

ASD is classified based on the location and morphology. Over 80% of patients with ASD have ostium secundum‐type defects located within the fossa ovalis and typically present with one or several defects.^104^, ^110^ The size of the defect varies and may reach more than 30 mm or result in a completely absent septum primum. Moreover, this type of ASD may enlarge with age and cardiac growth.^111^ Other types of ASD include ostium primum (approximately 15% of ASD), sinus venous (5% of ASD, including superior (90%) and inferior sinus venosus defects), and coronary sinus defects (less than 1% of ASD) (Figure 4).^104^, ^105^ ASD may also be a component of more complex CHD, for example, TOF and Ebstein anomaly.

Among CHDs, ASD has the highest increase in the incidence in recent years, which may be due to the widespread use of TTE and TEE.^2^ The incidence of ASD combined with PDA and VSD contribute to 93.4% of the increased overall prevalence of CHD.^2^ Interestingly, the incidence of ASD is negatively correlated with gross national income, suggesting that ASD occurs more frequently in regions with lower national incomes.^2^ This may be due to environmental and/or generic risk factors for ASD in lower‐income regions. Exposure to environmental conditions such as parental alcohol consumption, smoking, and antidepressant drug use has been documented to enhance the risk of having offspring with ASD.^7^

The risk of the transmission of ASD to offspring is 8−10%.^112^ A genetic predisposition to ASD has been reported and several generation mutations involved its pathogenesis, such as NKX2‐5/CSX, TBX5, and so on.^112^ The precise regulation of gene expression is central to embryonic development. Recent genetic studies have indicated that a region of chromosome 4p16, IncRNA STX18‐AS1, is associated with an increased risk of ASD. The deficiency of this region blocks cardiac lineage specification from the cardiac mesoderm into cardiac progenitors and cardiomyocytes and may inhibit heart development.^113^

#### 3.1.2 ASD‐associated complication

Major arrhythmias are far less common in children with ASD. However, atrial flutter and fibrillation (AF) are relatively common in patients with ASD, as the most frequent outcome of ASD.^114^ The duration of right atrial overload due to L‐R shunting rather than age per se results in time‐course‐related atrial structural remodeling, which may account for the occurrence of atrial arrhythmia.^115^ Patients with ASD exhibit an increased risk of new‐onset AF and hospitalization for AF than a control population.^114^ Patients who had undergone ASD closure still exhibited an unexpectedly high incidence of AF over long‐term follow‐up periods.^116^ Therefore, AF is a real issue of ASD regardless of whether the defect has been closed.^116^ The occurrence of new‐onset AF may lead to clinical deterioration in patients with ASD, which may be due to the decreased ability to tolerate the suboptimal hemodynamics of AF because of the L‐R shunt in these patients.

Patients with untreated ASD can develop PAH and irreversible pulmonary vascular disease because of a longstanding, hemodynamically significant shunt.^107^, ^108^ A nationwide population analysis reported that the prevalence of PAH was 7.4% in patients with CHD with systemic‐to‐pulmonary shunts.^117^ The pathophysiology of PAH secondary to ASD is variable and complex and affects management decisions. In addition, PAH is an independent predictor of prognosis in ASD, which limits the functional capacity and survival of patients.^117^ Prevention of PAH progression is the primary target of ASD therapy. Previous studies indicated that the development of PAH is associated with the type of ASD, in which sinus venous‐type ASD is more frequently associated with PAH than secundum‐type ASD and PAH occurs at a younger age in patients with sinus venosus‐type ASD.^111^ Severe PAH may ultimately result in the reversal of the shunt from right to left, causing cyanosis and Eisenmenger syndrome, though this is a rare complication. The evaluation of the pulmonary arterial pressure is critical in patients with ASD, especially prior to ASD closure.

### 3.2 Diagnosis of ASD

#### 3.2.1 Echocardiographic evaluation

In a multicenter cohort study, Dehn et al.^106^ reported that ASD was associated with morphological cardiac changes in neonatal age and that larger right atrial and ventricular dimensions were observed within a short time of L‐R shunting. Moreover, the left atrial volume was bigger in neonates with ASD than in those without.^106^ Therefore, the early detection of ASD allows for timely decision‐making and improves cardiovascular outcomes.

Currently, TTE is the primary technique for the detection and quantification of ASD.^118^ TTE can provide essential information regarding the anatomy, size, and location of the ASD; the degree and direction of shunting; and the pressure and flow through the pulmonary circulation.^119^ Importantly, TEE is invasive and no general anesthesia is necessary for most patients.^119^ In addition, the right atrial end‐systolic volume (RAESV) and tricuspid annular plane systolic excursion (TAPSE) can be assessed using TTE, which are markers for assessing right heart function. Although no association between ASD size and increased right atrial and ventricular volumes has been reported, a slight positive association was observed between ASD size, RAESV, and TAPSE.^106^ A careful TTE evaluation is necessary for selection of patients for transcatheter device closure.^120^ A retrospective study was performed on 52 patients with ASD who received transcatheter closure, and the results indicated that the preoperative right ventricular end‐systolic volume index (RVESVI) is an independent predictor for normalization of RV volume and that closure prior to an RVESVI of 75 mL/m^2^ may provide optimal timing for normalizing the RV volume.^121^

**FIGURE 5:**
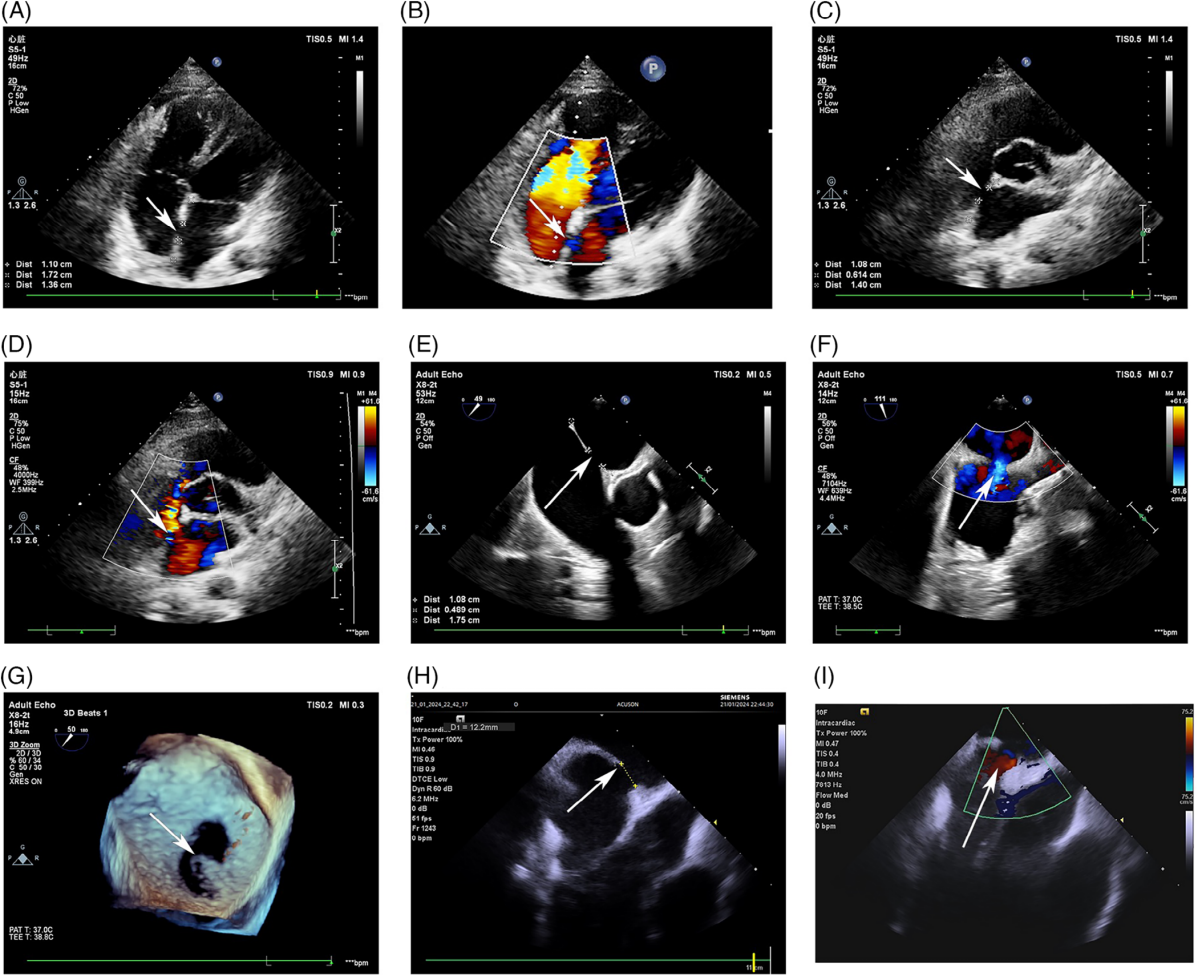
Echocardiographic images of atrial septal defects (ASDs). (A) Two‐dimensional transthoracic echocardiography (TTE) imaging of ASD in four‐chamber view. (B) Color Doppler flow imaging of ASD in four‐chamber view. (C) Two‐dimensional TTE imaging of ASD in subcostal view. (D) Color Doppler flow imaging of ASD in subcostal view. (E) Two‐dimensional transesophageal echocardiography (TEE) imaging of ASD. (F) Transesophageal color Doppler flow imaging of ASD. (G) Transesophageal three‐dimensional echocardiographic imaging of ASD. (H) Intracardiac echocardiography (ICE) imaging of ASD. (I) Intracardiac color Doppler flow imaging of ASD. Arrows point to the ASD.

Unfortunately, TTE sometimes cannot acquire the necessary imaging in some individuals due to poor transthoracic windows related to a big body habitus or previous thoracic surgeries.^105^ In addition, TTE imaging does not provide a comprehensive visualization of the interatrial septum.^111^ Therefore, the image quality of TTE is inadequate for the precise evaluation of the location and size of the defect, which may hinder referral for therapy. Sinus venosus‐type ASDs are typically associated with partial anomalous pulmonary venous drainage of the right upper pulmonary vein, which affects the ability of the TTE to adequately evaluate these defects.^112^ In addition, a coronary sinus ASD is difficult to visualize using TTE. TEE can be used to characterize the septum and provide an excellent image of ASD as it more closely examines the interatrial septum and is useful in individuals with unsatisfactory transthoracic windows.^122^ TTE offers better images of the margins or rims of the ASD and allows for an evaluation of the surrounding structures.^111^ An accurate identification of the margins and the surrounding rims of tissues and the associations of the ASD to the venae cava, coronary sinus, mitral valve, and tricuspid valve are critical for the assessment of the patient's candidacy for transcatheter closure. At present, TEE has been considered as a gold standard imaging modality for assessing ASD and is widely used in clinical settings. Moreover, TEE is superior to TTE in terms of device size selection. Intraoperative TEE can provide real‐time implant guidance and postdeployment evaluation of ASD closure. However, TEE for guiding ASD closure requires general anesthesia and is associated with the risks of laryngospasm, esophageal trauma, and aspiration pneumonia. Some individuals cannot tolerate TEE. In one retrospective study, there were no obvious differences of the incidence of long‐term complications, cardiac chamber sizes, or tricuspid regurgitation among individuals with ASD who underwent TTE or TEE, suggesting that TTE may be as safe and effective as TEE in assessing and guiding ASD closure in adults with lower body mass indexes.^123^ However, this previous study was a single‐center, retrospective study and sample population was small. Therefore, the selection bias cannot be excluded. A large‐scale, multicenter, and prospective study is required in the future. In the past decade, the application of three‐dimensional‐TEE has increased due to its particular advantage of providing an en‐face view of the anatomical characteristics of the ASD and unique views for the determination of the shape and changes in size of the defect during the cardiac cycle.^120^ In addition, three‐dimensional‐TEE imaging can be used to evaluate the relationship between the ASD and the surrounding cardiac structures, which can be used for real‐time guidance during device placement (Figure 5).^111^

#### 3.2.2 Advanced imaging techniques

A recent study reported a novel deep learning model applicable to color Doppler echocardiography for the automated evaluation of ASD that can enhance the reliability and validity of echocardiography for the screening and quantification of ASD.^119^ In recent years, four‐dimensional flow cardiovascular magnetic imaging has been used to assess ASD, overcoming the limitation of two‐dimensional phase‐contrast sequences and providing an excellent qualitative and quantitative evaluation of multifenestrated ASD.^110^ Although cardiac computed tomography (CT) and MRI are rarely required, they are considered complementary imaging modalities in some patients as they allow for the evaluation of extracardiac structures, measurement of right ventricular volume and function, quantification of the pulmonary‐systemic flow ratio, and identification of sinus venosus defects.^104^, ^112^ In addition, cardiac catheterization is necessary in patients with an increased pulmonary atrial pressure.

Over the last decade, intracardiac echocardiography (ICE) has been used increasingly to diagnose and treat ASD (Figure 5). ICE provides a clear view of the cardiac structures with superior spatial and temporal resolutions as the probe has large matrix arrays.^124^ The near‐field nature of ICE from the right atrium and the lack of structural interference allows for the avoidance of intracardiac acoustic shadowing when imaging the atrial septum.^124^, ^125^ ICE has been an alternative imaging technique to TEE for the morphological assessment of ASDs.^125^ During ASD closure, ICE does not require general anesthesia and minimizes the sedation requirements. Rigatelli et al.^126^ reported that ICE‐guided ASD closure without the use of sizing balloons is effective and safe, with very few procedural and late complications, including those in very‐long‐term follow‐up periods. Therefore, ICE has the potential to be the leading imaging technique for guiding ASD closure and monitoring procedure‐related complications. Several studies have indicated the superiority of ICE over TEE for guiding the closure of ASD.^127^, ^128^ However, the ICE operator must be experienced, and the catheters used for ICE are expensive. To date, few prospective, randomized controlled, large‐scale studies have comprehensively compared the effects of ICE and TEE for the diagnosis and treatment of ASD. Despite the development of newer techniques, TEE and TTE are still the key imaging modalities for the evaluation of ASD and guidance of ASD closure. ICE should be considered a complementary imaging modality.

### 3.3 Treatment of ASD

#### 3.3.1 Traditional ASD closure

The management of ASD is based on anatomical information, the magnitude of shunting, and the presence or absence of PAH. Several therapeutic options exist based on the ASD anatomy and patient's complications. A small ASD without right ventricle volume overload and other complications can be monitored, whereas ASD closure is recommended in the presence of a hemodynamically significant shunt that triggers the dilation of right heart structures, regardless of the patient's symptoms.^108^ ASD closure enhances patient survival and prognosis when performed at a young age. Muroke et al.^109^ reported that ASD closure prior to the third decade is not correlated with increased mortality.

During surgical operation, the ASD is directly visualized and closed. The first transcatheter ASD closure was performed in 1976.^129^ Transcatheter closure of an ASD using an occluder device has been approved as an effective and safe procedure in both children and adults. For most cases of secundum‐type ASD, transcatheter closure is preferred over surgery due to several advantages including the avoidance of surgical incisions and cardiopulmonary bypass, less invasive procedures, a shorter procedure time, fewer procedure‐related complications, a faster recovery, and shortened hospital stays.^130^ However, this procedure is associated with embolization, arrhythmia, cardiac tissue erosion, hemopericardium, infective endocarditis, delayed breakdown of the device, and other complications.^130^ Although the incidence of complications associated with transcatheter closure are rare, the consequences of the complications can be severe. Device embolism occurs in approximately 0.1–1.3% of patients due to an undersized device, absent posterior rim, large defect (>30 mm), or inadequate device placement.^130^, ^131^ Embolism can occur in the great vessels (including the aorta and pulmonary artery) and heart chambers and may be life‐threatening or need surgical intervention.^130^ After device implantation, antiplatelet therapy is necessary for a minimum of 6 months. Transient atrial tachyarrhythmia always occurs after the intervention.^4^ Cardiac erosion, hemopericardium, infective endocarditis, and delayed device breakdown occur rarely. In addition, approximately 15% of patients with ASD have new‐onset or worsening migraines after ASD closure. However, the pathogenesis of new‐onset migraine after ASD closure remains unclear. The residual shunt, incomplete endothelialization of the occluder, release of inflammatory mediators, and microthrombus formation may cause the occurrence or worsening of migraines after ASD closure.^132^, ^133^ In some patients, the migraines are associated with a nickel allergy, as nickel is a component of some ASD closure devices.^133^ Antiplatelet treatment may help ameliorate the migraines.^108^

Increased mortality due to ASD was not observed in patients who received transcatheter ASD closure in previous studies.^109^ The risk of long‐term outcomes were not different between patients who received transcatheter ASD closure and a control group and no distinction in all‐cause mortality or cumulative probability of cardiovascular mortality were observed.^114^ Therefore, transcatheter ASD closure is effective for improving the outcomes of patients with ASD. Moreover, transcatheter ASD closure improves cardiac remodeling, functional capacity, pulmonary pressure, tricuspid valve regurgitation, and plasma brain natriuretic polypeptide (BNP) levels in patients ≥60 years of age.^134^ ASD closure is available and encouraged in this older population based on the symptomatic benefits and improved quality of life.^122^ Recently, Suzuki et al.^135^ reported a 92‐year‐old man with decompensated heart failure who achieved excellent clinical outcomes after successful transcatheter ASD closure. Therefore, transcatheter closure may be an effective therapy even in very elderly patients with ASD. In other words, patients with ASD of any age can benefit from transcatheter interventions. However, older patients often have comorbidities and frailty. Residual functional tricuspid regurgitation and persistent right heart enlargement are more common in elderly patients with late ASD closure and are associated with increased BNP levels and a dysfunctional right ventricle.^122^ In a retrospective cohort study of 1290 adults with ASD who underwent transcatheter closure, older patients (>60 years) had a higher risk of all‐cause and cardiovascular mortality than adults under the age of 40 years.^114^ The prevalence of new AF was nearly 5‐fold increase in patients aged over 60 years than in younger patients. Therefore, ASD closure requires a careful risk–benefit evaluation in elderly patients.

Previously, ASD closure was contraindicated and harmful in adults with ASD with a significant reverse shunt (R‐L flow) or severe PAH. However, in February 2021, an ESC statement regarding the management of adult CHD indicated that when a significant L‐R shunt is present (flow ratio > 1.5), ASD closure is feasible when the PVR is between 3 and 5 wood units (WU). When PVR is greater than or equal to 5 WU, fenestrated ASD closure may be conducted if the PVR decreases to <5 WU following a PAH‐targeted therapy and the L‐R shunt remains evident.^4^ However, ASD closure should be avoided in patients with persistent PVR ≥ 5 WU or Eisenmenger syndrome despite adequate mediation, as noted in the ESC guidelines.^4^ Therefore, an appropriate measurement of PVR is important in the management of patients with ASD.^11^ Recently, a meta‐analysis of ten studies including 207 patients with ASD and severe PAH reported that ASD closure improves the hemodynamic and functional parameters with a low risk of major adverse cardiac events. According to the therapeutic options, the results showed that drug therapy to lower PAH first (treat‐and‐repair technique) was superior to direct ASD closure (straight‐to‐ repair technique) in these patients.^107^ Therefore, the feasibility of closure in these patients with ASD and severe PAH were highlighted. However, it also emphasized that closure of ASD in patients with severe PAH required careful evaluation to verify the reversibility of PAH prior to closure. As this was a retrospective study and the sample size was relatively small, a selection bias may be present. Therefore, additional prospective, large‐scale, observational studies are required.

#### 3.3.2 Advanced transcatheter closure of ASD

In patients with nonsecundum ASD, multiple ASDs, extremely large defects, a lack of sufficient margins and rims to safely anchor the device, or excessively bulging ASAs, transcatheter closure of the ASD is not optimal, and surgical repair is necessary.^108^, ^122^ However, the management of ASD has advanced over the past decade, resulting in improved therapy in some patients. Sinus venosus ASD is characterized by an anomalous systemic connection of one or more pulmonary veins, which is associated with a hemodynamically significant shunt. Surgery is the traditional therapeutic approach in these patients.^136^, ^137^ Recently, transcatheter closure via covered stent placement in the superior vena cava has been established as an alternative therapeutic option for some patients. A covered stent was used to replace the deficient posterior wall of the superior vena cava (SVC) and completely close the ASD.^138^ Those patients with a left SVC are always good candidates for this type of intervention.^138^ Several studies have proven the efficiency of this approach in recent years.^139^, ^140^, ^141^ However, these were retrospective studies with small sample sizes. This procedure is complex and may result in complications, including stent instability and potential migration, residual shunting, and embolization.^142^ In one retrospective study by Sivakumar et al.,^139^ 24 patients received transcatheter closure, including four (16.7%) who developed major complications such as stent migration (*n* = 3) and left innominate vein occlusion (*n* = 1). In another study of 75 patients who underwent stent implantation, 5.5% had major complications (including stent embolization, pericardial tamponade, and occlusion of the right upper pulmonary vein) and 60% experienced residual shunting. In addition, 44% of patients required additional stent implantation to anchor the stent or close the residual shunts.^140^ Therefore, technical modifications are needed to address stent migration, achieve complete coverage of the defect, and lower the risk of embolization. After stent implantation, patients require 6 months of antiplatelet therapy.^141^ In a small retrospective study, 14 patients underwent transcatheter repair of sinus venosus‐type ASD using the suture technique. No stent migration, stent embolization, arrhythmia, or severe complications were found, and all of the cases had clinically significant reductions in the right heart size. Moreover, no patient required reintervention after a mean follow‐up period of 16.5 ± 10.5 months. Only three cases in the previous study had a tiny residual shunt at the ASD level.^141^ However, this was a retrospective, nonrandomized study that included a small cohort and no comparative group; additional randomized studies with longer follow‐up periods would be required to confirm the superiority of covered stent implantation over the surgical approach in the management of these patients. Another recent study of 12 patients with inferior sinus venosus‐type ASD who underwent successful transcatheter closure using PDA occluders and three‐dimensional‐printed heart models reported that interventional therapy may not be contraindicated in patients with inferior sinus venosus‐type ASD.^143^ However, this previous study was a retrospective study with a small sample size, and prospective, randomized controlled trials including a large cohort of patients are necessary. At this time, the surgical repair remains the standard treatments in patients with sinus venosus‐type ASD.

Patients with coronary sinus‐type ASD have partial or complete unroofing of the tissue separating the coronary sinus from the left atrium. Surgery is the mainstay of treatment for these patients.^108^ The percutaneous closure of this defect is not feasible due to the complex anatomy of the septal fenestration and the risk of coronary sinus obstruction caused by the occluding device. In the past decade, few studies regarding the successful transcatheter treatment of unroofed coronary sinuses have been reported. However, these are sporadic case studies. In 2013, Santoro et al.^144^ first reported an 8‐year‐old patient with a partially unroofed coronary sinus who underwent the successful implantation of an Amplatzer septal occluder device. Wang et al.^145^ reported successful implantation using the Amplatzer septal occluder device in nine patients with coronary sinus‐type ASD. No residual shunt was found among all patients during the 3‐month follow‐up period, and symptomatic improvement was displayed in most patients after a mean follow‐up period of 42.6 ± 18.3 months.^145^ Recently, Zhou et al.^146^ presented a 50‐year‐old women with an unroofed coronary sinus defect, an enlarged right ventricle, and moderate tricuspid regurgitation who received a PDA occluder. After an additional 3‐month of follow‐up, the patient's right ventricle size normalized and the tricuspid regurgitation was alleviated.^146^ Therefore, transcatheter treatment for coronary sinus‐type ASD may be feasible in some patients. However, this procedure requires caution, and a detailed assessment of the local anatomy before and after intervention is necessary.

## 4 VENTRICULAR SEPTAL DEFECT

### 4.1 Pathophysiology of VSD

VSD is characterized by a hole or pathway between the ventricular chambers, which is one of the most common CHD. The prevalence of VSDs varies within populations and is determined by the sensitivity of the diagnostic method and the age at examination. The prevalence of VSDs in newborns is up to 5% based on population screening using highly sensitive color Doppler echocardiography.^147^ The majority of newborns with VSDs have tiny muscular defects that heal in the 5 year. VSD is present in up to 3−3.5 per 1000 live births.^147^ CHD screening among school‐aged children in the Qinghai Province revealed an overall prevalence of 6.73%, and VSD accounted for 9.9% of all CHD.^148^

VSD ranges in size from tiny pinholes to nearly the entire ventricular septum, and classification of VSD remains controversial. Most classification systems focus on the location of the defect or on the nearby anatomic structures surrounding the defect. The International Society for Nomenclature of Pediatric and Congenital Heart Disease provides a classification system in which VSD is divided into perimembranous central, inlet, trabecular muscular, and outlet VSDs based on the geography and borders of the defect.^149^

Perimembranous central VSD is located in the membranous portion of the interventricular septum and opens to the right ventricle at the center of base of ventricle. Typically, these defects are located below the commissure between the right and noncoronary leaflets of the aortic valve and behind the tricuspid septal valve. The fibrous structure between the atrioventricular and aortic valves usually participants in the one margin of the perimembranous central VSD. The leaflets of aortic valve may prolapse to right ventricle through the defect, resulting in aortic regurgitation.^150^

Inlet defects are located below the anteroseptal commissure of the tricuspid valve, extending along the septal leaflets. Inlet defects are divided into inlet muscular and inlet perimembranous defects. Inlet muscular defects have muscular borders, and the conduction system lies neighbor to the superior border. The anterosuperior border of the defects is the fibrous structure between the atrioventricular and aortic valves.^150^ And the posteroinferior rim is proximal to the conduction system. This type of defects is sorted by the exists of atrioventricular septal malalignment. Defects with septal malalignment are always coexist with overriding tricuspid valves or supero‐inferior ventricles. In these defects, the atrioventricular node is inferior to the position where the muscular ventricular septum infuses the right atrioventricular groove and the conduction pathway courses along the posteroinferior rim.

Trabecular muscular defects are located in the muscular ventricular septum. Some include multiple defects with different entrances and exits on each side. Trabecular muscular defects are further sub‐classified according to geographic location into midseptal, apical, posteroinferior, and anterosuperior types. Midseptal muscular defects are located within the middle of the apical muscular septum, which is different from the central perimembranous defects locating at the base of the ventricular mass. The apical muscular defect is located at distal of the moderator band. The posteroinferior muscular defect is located near the diaphragmatic part of the right ventricle, whereas the anterosuperior muscular defect is located anterior to the septal band.

Outlet defects are located within the right ventricular outlet and usually between the limbs of the septal band. The outlet defects are subdivided into outlet perimembranous, outlet muscular, and doubly committed juxta‐arterial defects. The posteroinferior rims of doubly committed juxta‐arterial defects are different in muscular or fibrous. The outlet perimembranous defect is close to the of the tricuspid anterior leaflet, whereas the central perimembranous defect is close to the septal leaflet. The outlet muscular defect is located above the posteroinferior limb, away from the atrioventricular conduction pathway. The doubly committed juxta‐arterial defect has a cranial border with a fibrous band between the pulmonary valve and aortic valve. This type of defects is accompanied with absent muscular outlet septum. The aortic regurgitation occurs when the right and noncoronary leaflets of the aortic valves prolapse into the defects. Outlet defects may be accompanied with the different types of outlet septum malalignment which typically occur with other CHDs.^151^

The pathophysiological response to VSD is determined by the flow of interventricular shunting and volume overload. The restrictive defects are intrinsically resistant to shunting. However, the shunting through larger defects is determined by the relative resistance of the pulmonary and systemic circulation. When PVR decreases, L‐R interventricular shunting increases and leads to excessive pulmonary blood flow. Large VSD without PAH are manifested as increased pulmonary blood flow and left heart volume overload which resulted in left heart dilation. The pulmonary pressure is variable and increases with age. Some patients with VSD may develop pulmonary hypertension in early adulthood or late childhood. Over time, the L‐R shunting flow begins to lessen and eventually reverses to the R‐L shunting, leading to Eisenmenger syndrome. The endothelial‐dependent vasoconstrictor and vasodilator pathways mediate the pulmonary vascular remodeling. The chronic elevation of the pressure promotes structural microvascular changes, including the migration of smooth muscle into nonmuscular vessels and the formation of plexiform lesions.

Other pathophysiological factors of VSD include aortic valve prolapse and obstruction of the pulmonary or systemic outflow tract. Defects located just beneath the aortic valve, such as doubly committed or perimembranous defects, can be complicated by aortic valve prolapse and regurgitation due to Venturi forces and the absence of structural support and commissural suspension of the leaflets.

The double‐chambered right ventricle is the formation of a proximal high‐pressure chamber and distal low‐pressure chamber within the right ventricle. The double‐chambered right ventricle mostly result from the outlet septum anterior deviation which is related to the hypertrophy of muscle bands. However, posterior deviation of the muscular outlet septum into the left ventricular outflow tract may result in muscular subaortic stenosis.

### 4.2 Diagnosis of VSD

A pansystolic murmur may be heard on clinical examination, indicating a high velocity of shunting through the VSD. Echocardiography is the predominant technique for diagnosing VSD, allowing for the detection of the defect size, location, and anatomical relationships to the valves and the associated prolapse of the aortic valve as well as the assessment of right ventricular pressure and loading on the right and left heart.^152^ Left ventricular hypertrophy resulting from volume loading of the left ventricle is noted on electrocardiography, and the right ventricular pressure is increased due to either PAH or right ventricular hypertrophy. TEE can be used to achieve an intraoperative assessment of VSD. Right heart catheterization is reserved for measuring the PVR in patients with pulmonary vascular disease.^153^

CMRI can offer specific information regarding the anatomy of patients with complex defects such as a double‐chambered right ventricle. CMRI allows for the quantification of stroke volume in both the right and left ventricles and extracardiac defects can be delineated clearly.^154^

### 4.3 Treatment of VSD

The 2020 ESC guidelines for the management of adult CHD provides recommendations for VSD interventions.^4^ VSD closure is recommended independent of the patient's symptoms when left ventricle volume overload occurs without PAH. These patients must have no signs of pulmonary artery pressure elevation or invasive confirmation of a PAP < 3 WU. VSD closure is also suitable in patients with a history of repeated infective endocarditis or VSD‐associated aortic valve prolapse with aortic regurgitation, even in the absence of an obvious L‐R shunt. In patients with PAH and a PAP between 3−5 WU, VSD closure should be considered when an obvious L‐R shunt is present. When the PAP ≥ 5 WU, the feasibility of VSD closure should be carefully evaluated per case. In patients with severe PAH or Eisenmenger syndrome who present with exercise intolerance, VSD closure is not recommended.

The closure approach is determined by the defect size, location, and history of vascular occlusions. Transcatheter VSD closure has become increasingly common, especially membranous VSD closure.^155^ Lower‐profile delivery systems and softer devices improve the success rates, with lower rates of conduction disturbances or heart blocks. The antegrade approach is the most commonly described technique for the closure of membranous, muscular, and postsurgical residual defects and postinfarction VSD. However, surgical repair is necessary in some patients who were not considered to be suitable candidates for transcatheter closure.

## 5 ATRIOVENTRICULAR SEPTAL DEFECT

### 5.1 Pathophysiology of atrioventricular septal defect

Atrioventricular septal defect (AVSD) accounts for about 4−5% of CHD and occurs at similar rates in male and female patients.^156^ AVSD is caused by the maldevelopment of endocardial cushions and characterized by the presence of a common atrioventricular junction. A complete AVSD includes a septal defect in the crux of the heart, involving both the interatrial and interventricular septa.^4^ Ostium primum ASD is also referred to as partial ASVD. Rarely, partial AVSD may have a defect only at the ventricular level. Over 75% of cases with complete AVSD have Down syndrome.^4^ AVSD can present on its own or in combination with complex CHD, for example, TOF.

The clinical manifestations of AVSD are dependent on the presence or size of the ASD and VSD, function of the left‐sided atrioventricular valve, and other associated cardiac defects.^4^ Symptoms are always nonspecific in that patients exhibit difficulty feeding, shortness of breath, dyspnea, exercise intolerance, and cyanosis, which is attributed to the magnitude of intracardiac shunting. Some patients with partial AVSD without other cardiac defects remain asymptomatic in early childhood and are diagnosed based on incidental findings. The prevalence of PAH is higher among patients with ASVD than among those with other CHD (34.4 vs. 3.2%).^117^

### 5.2 Diagnosis and treatment of AVSD

Approximately 50% of patients with untreated AVSD die during infancy. Therefore, a prompt diagnosis of AVSD in the fetus or newborn is essential to prepare for early surgery and reduce AVSD‐associated mortality. Echocardiography is the predominant diagnostic method for AVSD and can reveal the anatomical and functional characteristics of the heart. In addition, three‐dimensional echocardiography is applied as a complementary imaging modality as it offers a realistic en‐face view of the atrioventricular junction and valve morphology. Advances in diagnosis have been beneficial for achieving optimal outcomes.

AVSD is treated surgically, and complete AVSD surgery is typically performed in patient aged 3–6 months.^157^ The long‐term survival of patients with AVSD has improved because of advances in the surgical procedures. The optimal surgical approach is important to reduce the postoperative complications and reoperation rates. The modified single‐patch (MSP) and double‐patch (DP) techniques are effective procedures in terms of postoperative outcomes and long‐term reoperation rates.^158^ A cohort of 819 patients who underwent complete AVSD repair were studied retrospectively, showing that there was no distinction of the overall and event‐free survival rates between patients who underwent the MSP technique and those who underwent the DP technique.^159^ However, an underlying selection bias may have been present in the previous study due to its retrospective nature.

Infants aged less than 3 months with complete AVSD present with early heart failure requiring intervention. Recently, a few studies have already tried to assess the efficacy of surgery in infants younger than 3 months of age. One study of 304 patients with AVSD who underwent surgical correction revealed no difference in the 30‐day mortality between younger (aged less than 3 months) and older (aged more than 3 months) infants, suggesting that surgical correction is well tolerated in younger infants when clinically necessary.^160^ Another study reported that 194 patients aged less than 3 months with complete AVSD had an early mortality rate for surgical repair and pulmonary artery banding of 3.3 and 18.6%, respectively, and survival rates at 20 years of 92.0 and 63.2%, respectively.^157^ These results suggest that surgical repair of complete AVSD in infants younger than 3 months resulted in more favorable outcomes than pulmonary artery banding. Thus, neonatal repair of complete AVSD in patients younger than 3 months can be achieved with excellent results and should be the preferred strategy.

Despite advances in surgical repair, some patients develop arrhythmias long after surgery.^156^ In one retrospective, multicentric cohort study of 391 patients with AVSD who underwent surgery, 98 (25.1%) patients presented at least one episode of atrial arrhythmia during a mean follow‐up period of 17.3 ± 14.2 years. Atrial arrhythmia is associated with pacemaker implantation and heart failure, which can have devastating outcomes among patients. Interestingly, intra‐atrial reentrant tachycardia and focal atrial tachycardia were the leading types of arrhythmias in patients less than 45 years. In patients more than 45 years, AF was the most common arrhythmia. These results suggest that age, number of cardiac surgeries, atrial dilatation, and moderate/severe left atrioventricular valve regurgitation are independently correlated with an increased risk of atrial arrhythmias in patients with AVSD and that AVSD type and age at repair are not.^161^ Therefore, clinicians should be aware of the occurrence of atrial arrhythmia despite the success of surgical closure.

## 6 PATENT DUCTUS ARTERIOSUS

### 6.1 Pathophysiology of PDA

The ductus arteriosus is responsible for maintaining fetal circulation, which transferring blood from the pulmonary artery to the aorta for systemic circulation. After birth, the pulmonary artery pressure reduced and the duct will be closed.^162^ PDA is the persistence of ductal patency after birth. As a common CHD, the incidence of PDA was approximately 0.03‐0.08% in term infants and 30−67% in very premature infants and women were affected two times as often as men.^163^, ^164^

The hemodynamic impact of the PDA is based on the degree of the shunting from the aorta into the pulmonary artery, which depends on the flow resistance and pressure gradient between the aorta and pulmonary artery. The flow resistance is affected by several factors, including the length, narrowest diameter, and shape of the ductus arteriosus. Over time, L‐R shunting can result a higher pulmonary fluid volume and promotes pulmonary edema, bronchopulmonary dysplasia, chronic lung disease, and PAH, especially in premature infants.^165^, ^166^, ^167^ Pulmonary vascular remodeling and accelerated PAH induced by persistently PDA ultimately lead to R‐L shunting and Eisenmenger syndrome.^168^ In addition, abnormal shunting also induces left heart volume overload and systemic hypoperfusion, which aggravate left ventricular remodeling and predispose the development of necrotizing enterocolitis, renal failure, and death.^167^

### 6.2 Diagnosis of PDA

Some patients with PDA have no clinical symptoms, while others have several symptoms due to heart failure or Eisenmenger syndrome, including growth retardation, feeding difficulty, shortness of breath, fatigue, and dyspnea. Atrial arrhythmias, especially AF, often occur in these patients. A continuous “machinery” murmur located at the upper left sternal border is typically heard on physical examination. If the shunting is relatively large, a diastolic rumble is audible at the cardiac apex and the left ventricular impulse is prominent.

Echocardiography is the mainstay for the diagnosis and characteristics of PDA.^4^ Two‐dimensional imaging displays the shape of the ductus, and color Doppler is a highly sensitive technique used to assess the presence of PDA and estimate the magnitude of shunting. However, the ductus arteriosus is not always easy to identify using color Doppler in patients with R‐L shunting or low velocity L‐R shunting. In these difficult cases, our group found multiplane TEE was valuable for delineation of the structure and shunting of PDA.^169^ CMRI, CT, and cardiac catheterization have also been used to detect and evaluate PDA.^4^

### 6.3 Treatment of PDA

The development of PDA closure techniques contained interventional device closure and surgical repair. In adult with left ventricle volume overload and no PAH, PDA closure is recommended regardless of symptoms. In addition, PDA closure also is feasible in patients with PAH (PVR between 3 and 5 WU) and obvious L‐R shunting.^4^ However, to these patients with Eisenmenger syndrome or lower limb desaturation during exercise, PDA closure is unsuitable.^4^ Interventional closure is the main strategy in adult patients, whereas surgery is only used in patients with a duct too large for device closure or unsuitable anatomy.^4^ Among children and adolescents, device closure of the PDA remains the primary strategy, while surgical operation is limited to patients who are not candidates for device closure.^170^ Both interventional device closure and surgical closure have achieved excellent results, with >90% complete closure rates and a low incidence of complications in adult and pediatric patients.^171^, ^172^

PDA is correlated with a higher morbidity and mortality in preterm infants. Several studies have explored the appropriate timing for pharmacological, interventional, and surgical methods to close the duct in preterm infants. However, there is no definitive approach to reduce morbidity and improve outcomes. Nonsteroidal anti‐inflammatory drugs (NSAIDs), for example, indomethacin, ibuprofen, and acetaminophen, have been used to treat PDA. The two most commonly used therapeutic strategies are the intravenous injections of indomethacin and ibuprofen.^173^ A 2018 systematic review and meta‐analysis reported that intravenous injections of standard doses of indomethacin or ibuprofen are associated with a lower likelihood of hemodynamically significant PDA closure than a high dose of oral ibuprofen. Placebo or conservative management did not significantly change the incidence of mortality, necrotizing enterocolitis, or intraventricular hemorrhage.^174^ A 2019 review reported similar results regarding the effectiveness of drug therapy in PDA closure; however, the incidence of necrotizing enterocolitis was lower when ibuprofen or acetaminophen were administered than when indomethacin was administered, and a prolonged course of indomethacin was associated with a higher incidence of necrotizing enterocolitis compared with a shorter course.^167^ This review emphasized that the prophylactic use of NSAIDs did not improve the composite outcome of death or moderate‐to‐severe neurodevelopmental disability.^167^ A recent study reported that intravenous indomethacin led to an increased rate of PDA closure than ibuprofen, but was at higher risk for the development of acute kidney injury.^174^ A multicenter, randomized and double‐blind study reported that early ibuprofen administration resulted in a higher incidence of closed or small PDA, though the risks of death or moderate/severe bronchopulmonary dysplasia at 36 weeks postmenstrual age were not lower than those associated with placebo.^175^

When PDA does not respond to conservative treatment or pharmacological therapy, surgical ligation or interventional device closure may be necessary. Surgical ligation is an established invasive treatment; however, it is associated with critical complications, such as postligature syndrome, and is an independent risk factor for bronchopulmonary dysplasia and poor neurodevelopmental outcomes.^176^ Interventional device closure is a novel and minimally invasive therapy. Recently, it has been applied safely and successfully in preterm infants, including in extremely low‐birth‐weight infants.^177^, ^178^ A recent meta‐analysis showed that transcatheter intervention had a significantly lower rate of all‐cause mortality and hemodynamic instability than surgical ligation, suggesting that transcatheter PDA closure in preterm infants is a safe and effective alternative to surgical treatment.^179^

## 7 COARCTATION OF THE AORTA

### 7.1 Pathophysiology of coarctation of the aorta

Coarctation of the aorta (CoA) is a common congenital cardiovascular malformation, accounting for approximately 6−8% of CHD.^180^ It is a major birth defect of the aorta, with an incidence of 3−4 per 10,000 newborns and a male‐to‐female ratio of 2:1.^181^ CoA is characterized by narrowing of the aorta and has been regarded as a part of generalized arteriopathy, which is often located at the insertion of the ductus arteriosus and involves the aortic arch or isthmus.^182^ CoA can present as an isolated defect or be associated with other congenital abnormalities, such as bicuspid aortic valve, VSD, or PDA.^183^ The clinical presentation of CoA is determined by the severity of the lesion, including the degree of narrowing and length of the narrowed arterial tissue. A narrowed aorta may prevent sufficient flow cross the coarctation site, influencing distal perfusion. Due to compensatory mechanisms, patients exhibit upper‐extremity hypertension to maintain adequate perfusion.^181^ Patients with CoA are predisposed to a series of cardiac complications, including premature CAD, ventricular dysfunction, aortic aneurysms, and cerebrovascular disease. Neonatal CoA in which distal perfusion depends on the flow of the pulmonary artery through the PDA into the descending aorta is considered critical.^181^ When the ductus arteriosus closes, these neonates become acutely ill due to aortic obstruction leading to extreme end‐organ hypoperfusion, ultimately resulting in acute heart failure and renal dysfunction.^181^ However, other patients are asymptomatic or have mild manifestations until adulthood.

The pathophysiological mechanisms of CoA are multifactorial and have not yet been fully elucidated. After birth, tissue ingrowth from the ductus arteriosus causes constriction when exposed to higher arterial oxygen concentrations, which is considered the most common pathogenesis of isolated CoA.^180^ However, Gong et al.^183^ reported that the preductal CoA type is more common in patients younger than 3 years of age. Therefore, the type of CoA differs in different patient populations. Endothelial dysfunction, abnormal elastic properties, and inflammation have been showed to be involved in the pathogenesis of CoA.^180^

The risk of CoA is increased in the offspring of pregnant patients with CoA, supporting the presence of heritablity.^184^ Mutations of several specific genes are involved the pathogenesis of CoA, including mutations of the NOTCH1 and FOXC1 genes.^185^ Deep learning and identifying the genetic information associated with CoA will enhance the accuracy of disease prediction and facilitate the prevention and management of CoA.

Chronic hypertension, which often occurs in patients with CoA, is a risk factor for preeclampsia. The maternal mortality is significantly higher in patients with a combination of heart disease and preeclampsia than in pregnant women without cardiovascular disease.^186^ In 2022, the ESC Registry of Pregnancy and Cardiac Disease study reported a maternal mortality of 3.5% in patients with heart disease and preeclampsia. All deaths associated with preeclampsia occurred during the postpartum period, and 50% were attributed to heart failure.^187^ A recent study reported that preeclampsia occurred in 20% of patients with CoA during their first pregnancy, suggesting an elevated risk of developing preeclampsia than in the healthy pregnant population.^186^

### 7.2 Diagnosis of CoA

Upper and lower extremity blood pressure screening is necessary for the management of patients with CoA. When the blood pressure of the upper and lower extremities differs by more than 20 mmHg, CoA is suspected.^188^ Several cardiovascular imaging techniques have been used to detect CoA. TTE with color Doppler is the standard method for the diagnosis of CoA as it is noninvasive, easy to access, and allows for the evaluation of cardiac structure and function, blood flow direction and velocity, and cardiac and valvular abnormalities. TTE exerts a pivotal role during the postintervention follow‐up period and in the clinical management of CoA.^183^ However, TTE is limited by the acoustic window, and the quality of image is dependent on the operator's technique. Cardiac computed tomographic angiography (CTA) and CMRI serve as complements to TTE and provide useful information for the assessment of the anatomical features of CoA, delineating the site and degree of narrowing of the aorta, and visualizing the surrounding structures in both children and adults. These imaging modalities may also help develop a therapeutic strategy and discern CoA‐associated complications, such as aneurysms and residual or recurrent stenosis.^189^ The article by Gong et al.^183^ showed that CTA has a higher diagnostic accuracy than TTE. Combining TTE and CTA may improve the preoperative diagnostic accuracy for CoA and associated cardiovascular malformations.^183^ However, the disadvantages of CTA, including exposure to ionizing radiation and contrast‐induced nephropathy, should not be ignored.

Despite advances in modalities, the prenatal diagnosis of complex CHD remains a challenge. The accuracy of fetal echocardiography is limited by poor acoustic windows. The contribution of fetal ultrasound screening to the timely diagnosis of CoA is low, and 27% of infants with missed CoA diagnoses died at a median age of 17 days in previous studies.^183^, ^190^, ^191^ Thus, there is an urgent need to develop a better screening method for the timely detection of this life‐threatening cardiac defect to decrease the morbidity and mortality in affected infants. Fetal CMRI has recently been used to evaluate the cardiac function and intracardiac and vascular anatomy of the fetus, complementing fetal echocardiography and aiding in therapeutic decisions.^192^ However, due to the complexities of fetal identification, long examination times, possible detrimental cardiovascular repercussions, and maternal claustrophobia during MRI, fetal CoA remains a diagnostic challenge. Therefore, more studies are needed to improve the diagnostic strategies to increase prenatal detection rates and determine the patient's risk so that appropriate therapeutic decisions regarding the management of fetal CoA can be made.

### 7.3 Treatment of CoA

In patients with CoA, complete relief of the mechanical constraints of the stenosis is the goal of interventions such as balloon angioplasty, stent placement, and surgery. Early and timely therapy can effectively reduce the risk of long‐term morbidity and mortality, which is crucial for good outcomes.

Although balloon angioplasty appears to be effective for the short‐term relief of obstruction, it is associated with a higher prevalence of aneurysm formation and recoarctation, with a restenosis incidence ranging from 5 to 20%.^193^ Aortic wall injuries caused by balloon angioplasty may contribute to aneurysm formation. In addition, recovery may be attributed to elastic recoil, especially in young patients.^180^ Therefore, balloon angioplasty is rarely performed in infants and children.

Stent placement is the first‐choice therapy in most patients with CoA as it significantly reduces the peak systolic pressure gradient. This procedure is minimally invasive, safe, effective, and associated with few procedural complications. Stent placement is associated with a lower incidence of hypertension and lower prevalence of aortic wall injury and restenosis.^194^, ^195^ As a result, stent placement is the preferred treatment for CoA when anatomically feasible. Surgical intervention may be appropriate for infants and children with CoA. Surgery is usually performed in patients with coexisting complex CHD who are not candidates for stenting.

Unfortunately, the prognosis of patients with CoA after repair is not always favorable. Many patients develop hypertension even after complete relief from CoA obstruction, which is associated with reduced long‐term survival.^181^, ^188^ Therefore, continued blood pressure screening is necessary and targeting them is important to improve the long‐term patient outcomes. However, medication is not recommended for preventing hypertension in normotensive individuals with a history of CoA repair. Patients with repaired CoA present with reduced long‐term survival compared with the general population, which may be attributed to the progression of CAD. Pickard et al.^196^ reported that patients with CoA experienced myocardial infarction 7.2 years younger and received coronary intervention 15.6 years younger than those without CoA. Moreover, patients are at risk of aneurysm formation, recoarctation, and reintervention, even when the initial surgery and transcatheter intervention are successful.

Patients with CoA continue to have a risk of premature mortality even after successful repair, which is associated with hypertension, CAD, aneurysm formation/rupture, and cerebrovascular accidents.^180^ Regular, life‐long follow‐ups and multidisciplinary management are needed to optimize the outcomes of individuals with CoA. Further studies are required to elucidate the underlying pathophysiological mechanisms of CoA and identify patients who are at a greater risk of developing cardiac complications.

## 8 TRANSPOSITION OF THE GREAT ARTERIES

### 8.1 Pathophysiology of transposition of the great arteries

Transposition of the great arteries (TGA) is a severe form of CHD arising from an embryological disturbance that is characterized by inappropriate connections of the great arteries to the ventricles. In these patients with TGA, the aorta connects with a morphological right ventricle and the pulmonary artery arises from the morphological left ventricle. TGA triggers a series of detrimental changes in cardiac physiology, in which deoxygenated blood flows into the systemic circulation and oxygenated blood continually arrives in the pulmonary circuit. TGA is one of the commonest cyanotic congenital defects in newborns and accounts for nearly 3% of CHDs and 20% of cyanotic heart diseases.^197^ Based on the absence or presence of other CHD such as VSD, left ventricular outflow tract obstruction (LVOTO), and CoA, TGA is classified into simple and complex types. Complex TGA has a worse prognosis than simple TGA. Unfortunately, the etiology of TGA remains unclear.

### 8.2 Diagnosis and treatment of TGA

The timely diagnosis and treatment of TGA are paramount. Patients with untreated TGA rarely survive to adulthood. Surgical correction may achieve definitive treatment of this CHD. Relatively satisfactory long‐term survival outcomes and quality of life can be obtained after a successful operation conducted in the neonatal period, especially during the first 2 weeks of life.^198^ However, adult individuals with a history of TGA repair experience complications despite surgical success. The morphological right ventricle supporting the systemic circulation remains after the surgical procedure.^199^ The anatomic right ventricle may not sustain the systemic circulation chronically, and the patient's clinical condition deteriorates over time.^200^ Therefore, some patients may present with a series of late cardiac complications, including heart failure, tricuspid regurgitation, and arrhythmia.^201^ Patients with TGA and systemic right ventricle (SRV) have increased hospitalizations and mortality because of heart failure. Moreover, the incidence of mortality and hospitalization for heart failure was not associated with the average age at baseline.^202^ The prognosis of patients with TGA has been investigated in several studies. One study reported that decreased left ventricular ejection fraction (LVEF) and right ventricular ejection fraction (RVEF), higher NT‐proBNP levels, and a New York Heart Association (NYHA) class ≥ II are associated with a poor outcome in patients with TGA and SRV.^202^ Tricuspid regurgitation is frequent in survivors and predisposes them to progressive worsening, while sudden arrhythmia and heart failure are the most common reasons for late death.^199^ Some patients may require placement of a ventricular assist device or cardiac transplant.

However, other studies have reported heterogeneous results regarding postoperative outcomes in patients with TGA. No association between SRV function and mortality was reported in patients with TGA in a study by Roos‐Hesselink et al.,^200^ and the independent predictors were atrial flutter at the first outpatient visit after the Mustard procedure and additional repair of VSD and/or pulmonary valve stenosis. These conflicting results might be explained by the different inclusion criteria, sample sizes, and follow‐up periods of the studies. In addition, the patients only underwent echocardiography to assess the SRV function in the study by Roos‐Hesselink et al.,^200^ and the quantification of SRV function based on echocardiography is limited. CMRI is the preferred technique for accurately evaluating the SRV function. Recently, Lewis et al.^203^ reported that CMRI parameters (including indexed right ventricular end‐diastolic volume, RVESVI, right ventricular mass, and RVEF) were associated with an increased mortality risk, need for a ventricular assist device, or cardiac transplant in 101 patients with TGA and a SRV who underwent TGA surgery.^203^ CMRI may contribute to the risk stratification of patients with TGA.

Patients with repaired TGA may display a pathological neo‐aortic root dilation and progressive neo‐aortic regurgitation, which are correlated with elevated late morbidity.^204^ Neurodevelopmental impairment and brain injury in children with TGA have received increasing attention. Hicks et al.^205^ emphasized the importance of focused language interventions in the early postoperative period due to language delay at 2 years of age. Lim et al.^198^ reported that impaired brain growth and slower language development may be associated with surgical repair beyond 2 weeks of age, which may be due to extended periods of cyanosis and pulmonary over‐circulation. However, conflicting opinions have been reported, as other studies found no impact on language development. A total of 809 patients who underwent surgical repair during the neonatal period were retrospectively reviewed, and the results showed that the mean neurodevelopmental scores at the age of 5 years, including cognitive, motor, and language development, were not significantly impaired.^3^ Due to the heterogeneity among these studies, including the study designs and the characteristics of the enrolled populations, additional prospective and randomized studies are expected to acquire more identical conclusions regarding the neurodevelopmental outcomes of patients with TAG, which may help guide early interventions in these patients.

Take together, discerning the risk factors associated with adverse outcomes and recommending individualized surveillance strategies for patients with TAG are important for improving long‐term outcomes.^3^ Clinical evaluations should be performed annually, regardless of the type of TGA prior to surgery.

## 9 CONGENITALLY CORRECTED TRANSPOSITION OF THE GREAT ARTERIES

### 9.1 Pathophysiology of congenitally corrected transposition of the great arteries

Congenitally corrected transposition of the great arteries (ccTGA) is a rare anomaly that accounts for less than 1% of all CHD. The anatomical congenital anomalies differ from those in patients with TGA. In patients with ccTGA, the right atrium connects to the left ventricle from which the pulmonary artery originates, and the left atrium connects to the right ventricle from which the aorta originates. Moreover, an abnormal cardiac position is common in patients with ccTGA, as dextrocardia was reported in 19% of fetuses in a previous study.^206^ In addition, ccTGA is always associated with other cardiac defects including VSD, tricuspid abnormalities, and pulmonary outflow obstruction.^206^ The pathophysiology of the disease course remains not entirely clear.

In patients with ccTGA who do not undergo corrective surgery, the right ventricle involves the systemic circulation and a SRV is established. SRV enlargement and dysfunction is common due to the progressive remodeling of the right ventricle in patients with ccTGA. Patients with ccTGA and SRV present an enhanced risk of heart failure and long‐term mortality, and heart failure is the most frequent reason of death in these patients.^207^ A total of 96 patients with ccTGA and SRV aged ≥16 years were enrolled and followed for a mean of 6.5 years, the results revealed that 10.8% of patients died and 51.6% were hospitalized due to heart failure.^207^ A meta‐analysis showed that the mortality rate among patients with ccTGA was 1.9 per 100 patients/year; however, no apparent discrepancy of mortality was observed between patients with ccTGA and those with TGA after atrial switch. Patients with ccTGA also had an increased hospitalization for heart failure than those with TGA after atrial switch (4.3 vs. 3.2 per 100 patients/year); however, the rates of ventricular and supraventricular arrhythmias were not different between them.^202^ A recent study reported that patients with ccTGA experience various stages of deterioration leading to end‐stage heart failure or death over time. The incidence of death significantly increased between the ages of 51 and 69 years.^208^ Therefore, the identification of patients who are most likely to rapidly progress towards heart failure and death is important.

Unlike those with TGA, some patients with ccTGA remain asymptomatic until adulthood. Therefore, ccTGA is often diagnosed later, which makes the SRV more susceptible to dysfunction, ultimately leading to heart failure.^202^ Progressive decreases in SRV function and tricuspid regurgitation are the main complications of ccTGA. In a previous study, 34% of patients with ccTGA had reduced SRV function, and this percentage increased to 46% after 6.5 years of follow‐up. SRV dysfunction has been recognized as a common cause of death in patients with ccTGA.^207^ In addition, patients with ccTGA and SRV have a higher incidence of tricuspid regurgitation than patients with TGA who underwent Mustard or Senning repair.^203^ Severe SRV impairment and tricuspid regurgitation have been reported as predictors of resurgery, though these correlations were not supported in following multivariable analysis.^207^ As these findings are from a single‐center study that included only 96 patients with ccTGA, the risk of bias cannot be ruled out. A more recent multicenter study of 558 patients with ccTGA revealed that patients with moderate/severe right ventricle dysfunction and tricuspid regurgitation had a higher risk of the composite primary outcome of mechanical circulatory support, cardiac transplant, or death.^208^ However, the patient's anatomy and the need for tricuspid valve surgery were not identified as predictors of the primary outcome.^208^

Improving the functional status and prognosis of patients with failed SRV remains a major challenge. Data regarding the prognosis of patients with ccTGA are limited, especially in adult patients. Therefore, additional studies are needed. Recently, an association between NYHA class and the clinical outcomes of patients with ccTGA has been reported. Auer et al.^207^ indicated that NYHA class ≥ III and decreased left ventricle systolic function are independent predictors of death based on the multivariable analysis. Few studies have focused on sub‐pulmonary left ventricular function in patients with ccTGA, which may provide a new direction for assessing risks in these patients. However, the studies were single‐center, retrospective, small‐sample studies; randomized controlled trials with larger populations are needed.

### 9.2 Diagnosis and treatment of ccTGA

Echocardiography and CMRI are the preferred techniques for the diagnosis of ccTGA. Timely prenatal detection and prediction of the clinical outcomes of fetal ccTGA are important. Tricuspid regurgitation and arrhythmia are common in fetuses with ccTGA. Patients with isolated ccTGA are more likely to have a fetal atrioventricular block than those with CHD, due to the abnormal disposition of the conduction system.^201^ Recently, Cohen et al.^206^ conducted a multicenter retrospective study of 205 fetuses diagnosed with ccTGA and defined the predictors of poor outcomes as mild or greater tricuspid regurgitation, arrhythmia, pulmonary atresia, aortic outflow tract obstruction, and worsening hemodynamics on serial echocardiograms. In the previous study, nearly 40% of newborns exhibited functional or anatomical changes. Progressive tricuspid regurgitation and pulmonary outflow obstruction were the most common functional changes, and the most common anatomical changes included the presence, size, and location of a VSD and the structure of the tricuspid valve.^206^ Therefore, serial fetal echocardiographic follow‐ups are necessary to assess the cardiac structure and progression of the hemodynamic function in fetuses with ccTGA. The presence of a small VSD or Ebstein anomaly should be carefully assessed when ccTGA is suspected. In addition, periodic evaluations are needed to determine the need for surgery.

## 10 CORONARY ANOMALIES

Congenital coronary anomalies are rare CHDs that include an anomalous aortic origin of the coronary artery (AAOCA), an anomalous coronary artery from the pulmonary artery (ACAPA), and coronary fistulae. Most coronary anomalies are thought to be benign. However, some anomalies have an increased risk of myocardial ischemia, exhibiting a wide spectrum of symptoms, for example, angina, AMI, and SCD. Coronary anomalies remain the second leading cause of SCD in young individuals.^209^

### 10.1 Anomalous aortic origin of the coronary artery

AAOCA is an anomalous coronary connection from the contralateral artery, inappropriate aortic sinus, or ascending aorta. An anomalous RCA is predominant in individuals with AAOCA.^210^ Most patients with AAOCA remain free of exercise restrictions. Molossi and colleague showed that 49% of patients with AAOCA presented with incidental findings.^210^ In another study of 220 patients, 168 (76%) did not experience exertional symptoms and only 52 (24%) had exertional chest pain or syncope.^209^ Notably, AAOCA is associated with myocardial ischemia and SCD. SCD may occur in asymptomatic patients during or shortly after exertion.^209^ However, the identification of patients who may be at a higher risk of ischemia and sudden adverse cardiac events remains unclear. Molossi et al.^210^ recently reported that the African American race, an older age at diagnosis, an intramural course, and syncope on exertion were predictors of high‐risk lesions, suggesting that these may be associated with SCD. However, the previous study was a single‐center study, and the classification of high‐ and low‐risk groups was arbitrary.^210^

Previous guidelines reported that asymptomatic individuals with AAOCA have a low risk of SCD and require no additional testing upon a normal exercise stress test.^211^ In a 2023 study of 220 patients <21 years with AAOCA, Doan et al.^209^ proposed that exercise stress was a poor predictor of ischemia. Therefore, the determination of patient risk based solely on this test should be conducted with caution. Stress perfusion imaging can be used to determine the rate of myocardial ischemia in these patients.^209^ However, this was also a single‐center study with a limited patient population, which may result a selection bias. Therefore, multicenter, large‐scale prospective studies with long follow‐up period should be conducted in the future to determine the features of patients with AAOCA who are at an increased risk of adverse events.

The accurate evaluation and management of patients with AAOCA are essential. Coronary CTA is the preferred technique for diagnosing AAOCA as it provides a precise description of the anatomy of the anomalous coronary artery. The CT‐derived fractional flow reserve (FFRCT) test is an emerging noninvasive method that can efficiently evaluate the anatomy and function of the overall coronary artery, which is beneficial for the management of patients with AAOCA. Prior studies indicated that the presence of AAOCA was associated with a moderate hemodynamic decrease in FFRCT. However, the FFRCT did not differ between the patients with AAOCA who were determined to be at‐risk and those who were not at‐risk.^212^

Surgery is an important therapeutic strategy for AAOCA based on the patient's clinical manifestations and risk stratification. However, the management strategies published by the AHA/ACC and ESC is not identical. The AHA/ACC guidelines state that patients with AAOCA who are symptomatic or have a positive stress test should undergo surgical repair, which is a class I recommendation. The ESC guidelines provide a class I recommendation for surgery in symptomatic individuals with a positive stress test or other abnormalities of high‐risk anatomy.^5^ More evidence is required to guide clinicians regarding the optimal selection of surgical candidates that may avoid unnecessary operations in low‐risk patients.

### 10.2 Anomalous coronary artery from the pulmonary artery

Most patients with ACAPA have a left coronary artery originating from the pulmonary artery. This disease is life threatening, and a majority of patients present heart failure and coronary ischemia early in life, with 90% of patients dying suddenly at an average age of 35 years.^213^ Therefore, the timely diagnosis of ACAPA is crucial. Echocardiography is the current mainstay of diagnosis for ACAPA. However, ACAPA cannot be fully identified via echocardiography. Demir et al.^213^ reported that coronary anomalies were clearly visualized in 57% of patients assessed using echocardiography. Therefore, cardiac CT, CMRI, and coronary angiography should be used in patients in whom the diagnosis of ACAPA via echocardiography is unclear.

Guidelines recommend surgery for the therapy of ACAPA. In a small retrospective study of 14 patients with ACAPA, respiratory distress and murmur were the most common symptoms, and eight patients diagnosed during infancy presented with heart failure symptoms. All patients underwent surgery. Compared with the preoperative value, LVEF significantly improved during the first postoperative month, and the LVEF was normal in all patients at 6 months of follow‐up.^213^

### 10.3 Coronary fistula

Coronary fistula is the most common type of congenital coronary anomaly. Coronary fistula is the abnormal connection of a coronary artery to a heart chamber or other vessel. In children, a coronary cameral fistula is the most common type of defect, whereas a coronary‐to‐pulmonary artery fistula is predominant in adults.^214^ Coronary fistula is classified according to the size relationship to the coronary artery not feeding the fistula: small (<1 times the diameter), medium (1–2 times the diameter), or large (>2 times the diameter).^215^ The majority of defects are isolated, though associations with other CHD, such as ASD, VSD, and TOF, have been reported.^214^ Although most individuals are asymptomatic and discovered incidentally via cardiac imaging, larger fistulas can present with symptoms due to marked shunting and progressive dilatation of the fistula and cardiac chamber, including angina, AMI, and heart failure.^215^ However, most patients with a prenatal or postnatal diagnosis of congenital fistulae have a good prognosis and do not require intervention.^216^

Echocardiography, cardiac CTA, CMRI, and catheterization have been used to comprehensively evaluate coronary fistulae.^214^ Spontaneous closure of the fistulae is rare. Small and asymptomatic coronary fistulae always do not require special treatment but should receive a regular outpatient follow‐up. Surgical repair and intervention are alternative therapeutic options for patients with large, hemodynamically significant coronary fistulae. Percutaneous intervention techniques can be used to achieve coronary fistula closure. Notably, complex coronary fistulae can also be treated via percutaneous intervention. Recently, Sumaya et al.^217^ used the telescope anchoring technique to close gigantic bilateral coronary fistulae in a 55‐year‐old man. Unfortunately, considering the anatomical variability, some patients still require surgical repair.

## 11 LVOTO AND AORTOPATHIES

LVOTO and aortopathies are a spectrum form of CHD, which is associated with an up to 100‐fold higher risk of SCD than that in an age‐matched control population.^11^ A previous meta‐analysis reported that the incidence of LVOTO is higher in regions with a higher mean national income.^2^ However, the prevalence of LVOTO is decreasing compared with those of milder CHD (such as ASD and PDA), which may be due to prenatal diagnoses and subsequent termination of pregnancy when these severe defects are diagnosed, especially in developed countries.^2^

### 11.1 Congenital AVS

AVS is one of the most common valvular heart diseases, accounting for 3−6% of cardiac malformations.^218^ Morphological manifestations of congenital AVS range from partially fused commissures to missing cusps, leading to a functional bicuspid or monocuspid valve.^219^ The clinical manifestations range from mild to severe, according to the aortic valve morphology and degree of AVS. AVS can block the left ventricular outflow tract, thus enhancing the left ventricular pressure. These changes promote myocardial hypertrophy and flow reduction through the left ventricle, which may induce the development of hypoplastic left heart syndrome (HLHS).^220^ In newborns with severe AVS, a left ventricular dysfunction and duct‐dependent systemic circulation will be rapidly developed when the disease is untreated, even the patient will die within the first week of life.^221^ Patients who survive to adolescence or adulthood often remain asymptomatic for several years. Approximately 10% of adolescent or adult cases experience symptoms of congestive heart failure, such as dyspnea.^222^, ^223^

Physical examination reveals a systolic ejection murmur at the second right intercostal space and electrocardiogram often reveals left ventricular hypertrophy, myocardial ischemia, and arrhythmia. The diagnosis of AVS depends primarily on echocardiography, which can clearly show the morphology of the aortic valve. In addition, Doppler echocardiography can be used to determine the peak aortic jet velocity and mean gradient and the aortic regurgitation. In newborns with severe AVS, echocardiography can be used to determine if biventricular repair or univentricular palliation should be conducted.^170^ Still, CMRI and CT may provide additional information about patient's aortic morphology and coronary anatomy.

In pediatric patients, AVS interventions are recommended in symptomatic or asymptomatic individuals with severe AVS characterized by a mean gradient >40 mmHg detected via noninvasive Doppler or a peak‐to‐peak gradient >50 mmHg.^170^ Treatment should also be considered in patients with moderate stenosis who have ST‐segment changes at rest, reduced left ventricular function, or left ventricular dilatation.^170^ Effective pharmacological treatments for congenital AVS remain unclear. In most patients, transcatheter or surgical repair is performed to alleviate AVS and delay valve replacement until they are needed. The 2020 ESC guidelines indicate that transcatheter aortic valve implantation is unsuitable in the therapy of congenital AVS, except in very few cases who are not surgical candidates.^4^ Currently, fetal aortic valvuloplasty (FAV) has been used in fetuses with AVS and HLHS. A recent study found that, if handled by experienced operators, FAV can achieve a high technical success rate with the ability to achieve biventricular circulation and a low rate of surgery‐related mortality.^224^ However, the long‐term outcomes of FAV require further investigation.

### 11.2 Supravalvular aortic stenosis

Supravalvular aortic stenosis is an obstruction of the ascending aorta at or above the sinotubular junction caused by the deletion or mutation of the elastin gene located on chromosome 7q11.23.^225^ Although isolated lesions can occur, it is often a characteristic feature of Williams‐Beuren syndrome.^226^ Supravalvular aortic stenosis results in an increase in the left ventricular pressure and causes the left ventricle to concentric hypertrophy. As the coronary artery ostia are located at the proximal end of the stenosis, this pressure gradient leads to insufficient blood supply to the coronary arteries, which may cause angina pectoris during childhood.^227^

The primary diagnostic method for supravalvular aortic stenosis is echocardiography, which can be used to evaluate the location, form, and severity; mean and maximum gradients; and structure and function of the left ventricle. CMRI and CTA are equally suited for investigating the aorta.^170^ Surgery is the mainstay treatment for this type of lesions. The operative mortality for fibrous diaphragms and hourglass deformities is less than 5% and the 20‐year survival rate of operative repair is 80−85%.^228^ However, an updated meta‐analysis reported that the 30‐year survival rate after surgical repair of supravalvular stenosis is lower than the matched general population survival, and the lifetime risk of reintervention is significant, suggesting the necessity of lifelong monitoring of the cardiovascular system, especially in patients with residual stenosis and coronary obstruction.^229^

### 11.3 Subaortic stenosis

Subaortic stenosis, also termed subvalvular aortic stenosis, is characterized by LVOTO below the aortic valve, and is often associated with VSD, AVSD, or Shone complex (multilevel left heart obstruction).^170^ Subaortic stenosis accounts for 8−30% of patients with congenital LVOTO and includes four anatomic forms: a thin discrete membrane composing of an endocardial fold and fibrous tissue; a fibromuscular ridge; a diffuse, fibromuscular, tunnel‐like narrowing of the left ventricle outflow tract; and accessory or anomalous mitral valve tissue.^170^, ^230^ The endocardial fold and fibromuscular ridge types account for 70−80% of patients with subaortic stenosis.^170^ Subaortic stenosis obstructs the left ventricle outflow tract, increases the left ventricular pressure and induces myocardial hypertrophy despite normal arterial blood pressure. Meanwhile, jet damage to the valve results in secondary aortic regurgitation.^231^ Even after a long period, patients with mild or moderate isolated lesions remain asymptomatic. While severe subaortic stenosis can result in exercise intolerance, dyspnea, chest pain, syncope, heart failure, and pulmonary edema.^232^

The primary diagnostic method for subaortic stenosis is echocardiography, which can effectively evaluate the anatomy of left ventricle outflow tract, degree of subaortic stenosis, and function of the left ventricle.^230^ Additionally, CMRI is useful for identifying and evaluating complex LVOTO anatomy, particularly in patients with poor acoustic windows.

Surgical repair is the only effective therapeutic strategy for the patients with subaortic stenosis. Surgical strategies include the removal of fibrous membranes or fibromuscular rings with or without myectomy and mitral valvuloplasty.^4^, ^170^ To tunnel‐type lesions, a wide excision or Konno procedure is advisable.^4^, ^170^ Surgical results are favorable, and surgical mortality is less than 1%. However, the recurrence rate of subaortic stenosis is significant, and progressive aortic regurgitation also occurs postoperatively.^230^, ^233^

## 12 RIGHT VENTRICULAR OUTFLOW TRACT OBSTRUCTION

### 12.1 The pathophysiology of RVOTO

RVOTO comprises a wide spectrum of anomalies, including sub‐infundibular, infundibular, pulmonary valvular, supravalvular, and pulmonary arterial defects, depending on the location of the obstruction. Of those, pulmonary valve stenosis occurs in up to 90% of patients with RVOTO.^8^ RVOTO is characterized by flow limitation from the right ventricle to the pulmonary arteries and an increased systolic pressure gradient between these regions. These defects can induce an elevation of right atrial pressure and right ventricular dysfunction and are associated with increased morbidity and mortality.^9^ Increased right ventricular outflow tract pressure can reduce blood flow into the pulmonary arteries, thereby affecting cardiac output, compromising coronary blood flow and predisposing the heart to ischemia and serious arrhythmias.^4^

RVOTO presents with diverse features, ranging from isolated lesions to other complex CHD, for example, VSD and TOF. However, studies regarding RVOTO have reported conflicting results. A meta‐analysis regarding the prevalence of RVOTO found that RVOTO can be present in a several types of CHD in the adult population, including 49% (30 out of 61) associated with hypertrophic cardiomyopathy, 25% (15 out of 61) with double‐chambered right ventricle, and 5% (three out of 61) with TOF.^9^ These findings may be attributed to the heterogeneous populations and reporting bias. Pulmonary valvular stenosis often presents as an isolated lesion, though sub‐infundibular lesions are typically associated with VSD and infundibular lesions are commonly combined with VSD and TOF.

The clinical presentation of RVOTO involves a variety of spectra based on the heterogeneity of the anatomical features, ranging from asymptomatic or minimally symptomatic (such as shortness of breath and chest discomfort) to dyspnea. The symptoms may progress over time, and patients with severe stenosis have poor outcomes. In patients with critical pulmonary stenosis, right ventricular hypertrophy is also present, which may lead to cyanosis.^8^ Moreover, the severity of right ventricular hypertrophy will change based on the greater degree of outflow obstruction.^8^

### 12.2 Diagnosis of RVOTO

Echocardiography is a reliable, noninvasive technique to accurately evaluate the exact position and level of RVOTO and assess the severity of the defect by measuring the flow velocities across the obstruction. The diagnosis of RVOTO depends on the values of a systolic pressure gradient between the main pulmonary artery and the right ventricular outflow tract, which is considered a hemodynamic instability when the maximal systolic pressure gradient exceeds 25 mmHg.^9^ When echocardiography is inconclusive, cardiac CT, CMRI, and cardiac catheterization are helpful for the diagnosis of RVOTO.

However, the diagnosis of the anatomical cause of RVOTO remains challenging as cardiovascular dysfunction is often not displayed at rest. As symptoms occur during exercise and the degree of RVOTO may vary during exercise, Santens et al.^234^ emphasized the role of exercise CMRI in the evaluation of the physiological characteristics of patients with RVOTO during exercise. These imaging findings will help guide clinical decision‐making beyond the echocardiography findings.^2^ However, the benefits of this imaging modality are based on a case study. Prospective randomized controlled trials with larger sample sizes are necessary.

### 12.3 Treatment of RVOTO

The therapy of RVOTO depends on the lesion and severity of the obstruction. When stenosis is severe, patients with RVOTO should receive intervention regardless of their symptoms.^1^ Mild pulmonary stenosis is innocuous, and no immediate treatment is needed. Transcutaneous balloon expansion is recommended for individuals with severe valvular pulmonary stenosis.^13^ This procedure is safe and efficient for patients in all age groups, even for neonates. However, 5−20% of patients experience recurrent valve stenosis and may require repeat expansion.^2^ When balloon expansion is anatomically unfeasible or unsuccessful, surgical repair is recommended.^13^ Surgical procedures are appropriate in patients with sub‐infundibular and infundibular defects or in those with comorbidities that require surgical intervention.

Patients with pulmonary atresia and an intact ventricular septum may undergo transcatheter pulmonary valve perforation to maintain a stable flow of pulmonary artery and relieve the obstruction.^2^ However, this procedure is associated with a high risk of complications and repeat valvuloplasty and surgical repair are common.

## 13 TETRALOGY OF FALLOT (TOF)

TOF is one of the most common cyanotic congenital heart lesions, accounting for 5−10% of newborn CHDs.^235^ Several factors are likely to lead to the development of TOF. NOTCH1 mutations are commonly associated with complex CHDs.^185^ Maternal alcohol consumption significantly enhances the risk of TOF in offspring, which differs from other specific congenital phenotypes.^7^

TOF has four anatomic features: right ventricular hypertrophy, VSD, overriding aorta, and RVOTO.^236^ The right ventricular outflow tract displaces a large VSD and obstructs the right ventricular outflow at the infundibular, valvular, or supravalvular levels. The degree of obstruction is varied widely, ranging from mild to complete pulmonary valve atresia with diminutive or absent branch pulmonary arteries. Surgery for TOF involves VSD closure and relief of the RVOTO to the greatest extent possible.^237^ Transcatheter pulmonary valve replacement has been developed in the last two decades and is a valuable nonsurgical alternative to restore the right ventricular outflow tract and right ventricular function and reduce patients’ lifetime risks related to surgery.^238^ Therefore, the risk of coronary artery obstruction should be assessed before stenting the conduit. Balloon dilation and pre‐stenting can relieve conduit stenosis and provide a suitable landing zone for the valves. The delivery system can then be sent to the appropriate position and the valve can be implanted via balloon dilation. Patient survival from reintervention after transcatheter pulmonary valve replacement are comparable to the outcomes of surgical conduit or valve replacement.^239^

## 14 EBSTEIN ANOMALY

Ebstein anomaly is an infrequent congenital heart defect characterized by the abnormal formation of the leaflets of the tricuspid valve. In this defect, the anterior leaflet of the tricuspid valve typically originates at the annular level, while the septal and posterior leaflets are displaced towards the apex of the right ventricle, which result in right ventricular atrialization.^4^ Consequently, the functional right ventricle is anatomically reduced and accompanied by tricuspid valve regurgitation.^4^ Right ventricular dysfunction and tricuspid valve regurgitation reduce the pulmonary forward flow, leading to volume overload and dilatation of the right heart system, which may cause chronically low systemic cardiac output and heart failure.^170^, ^240^ The disease might be clinically silent, or have manifested itself in several symptoms, such as arrhythmias, dyspnea, fatigue, chest pain, and cyanosis. Complications of Ebstein anomaly mainly include high‐grade tricuspid valve regurgitation, right ventricle dysfunction, heart failure, hepatomegaly, paradoxical embolism, pulmonary embolism, tachyarrhythmia, infective endocarditis, and SCD.^237^

Echocardiography is the key diagnostic imaging technique for this defect as it provides information regarding the anatomy and function of the tricuspid valve.^4^, ^170^ In addition, color Doppler echocardiography allows for the quantification of tricuspid valve regurgitation. In addition, chest radiography, CMRI, and CT can provide information regarding the cardiac structure and function in patients with Ebstein anomaly.

The patient's clinical manifestations determine the treatment strategies for Ebstein anomaly. Signs and symptoms such as cyanosis, heart failure, and arrhythmias are principal indications for therapy. Treatment should be considered in asymptomatic patients who have a decreased working capacity and peak oxygen uptake. Pharmacological treatments, including intravenous prostaglandins in cyanotic neonates, diuretics to reduce the right ventricular preload in patients with heart failure, and antiarrhythmic drugs, focus on symptom improvement. Interventional treatments include catheter ablation of the right‐sided accessory pathway or the slow pathway in symptomatic individuals with tachyarrhythmia and atrioventricular node reentry.^4^, ^170^ Surgery continues to be a challenge and should only be performed by skilled, experienced surgeons. Repair of the tricuspid valve is preferred to its replacement. In addition, plication of the atrial right ventricle, closure of the septal defects, and repair of the associated lesions have been performed.^241^ Right ventricular unloading using a bidirectional cavopulmonary shunt is performed to repair advanced Ebstein anomaly with severe right ventricle dilatation and dysfunction.^242^ Heart transplantation may be the only potentially therapy for cases with severe biventricular dysfunction or failed repair.^4^

## 15 CONCLUSION AND PERSPECTIVE

CHD is an important component of cardiovascular disease and affects millions of newborns annually. Despite significant improvement in the quality of life and prognosis of patients with CHD following technological advancement, the exact etiology of most types of CHD remains unclear, and prevention of CHD continues to be a great challenge. In addition, several clinical issues need to be resolved.

First, as the most common type of CHD, PFO‐mediated paradoxical embolism may lead to stroke, AMI, and occlusion of the retinal, renal, peripheral, and mesenteric arteries. Thus, a thorough cardiac evaluation of patients with embolic ischemia is of paramount importance. However, whether PFO is a causative mechanism of ischemic stroke or other systemic embolisms is often indefinite. Thus, it remains a challenge to distinguish incidental from pathogenic PFO in many patients with paradoxical embolism. In addition, although there is usually a defined mechanism of stroke in patients aged >60 years, such as atherosclerosis and atrial fibrillation, PFO should not be ignored as a potential source of paradoxical embolism in these patients. Elderly patients can also benefit from PFO closure through a carefully selection.

Second, many mild CHDs, including asymptomatic PFO, tiny ASD, or VSD, may not lead to dismal outcomes and thus do not require special treatment. However, in patients with moderate or severe diseases, such as TGA, TOF, or severe CoA, timely diagnosis and treatment are necessary to improve the prognosis of these patients. Patients with untreated complex CHD may die soon after birth due to complications and it has been documented that prenatal diagnosis can decrease CHD‐associated neonatal mortality.^243^ Until now, prenatal echocardiography remains the main screening tool, though it has not been applied widely in pregnant women. Therefore, establishing a thorough screening program during the perinatal period may help identify cardiac malformations. In addition, development of advanced technology in fetal examination, including CMRI and artificial intelligence, may further improve the capacity of early and accurate detection of CHD in the future.

Third, due to the advances in medical technology, the majority of patients with CHD may survive to adulthood. Nonetheless, the influence of CHD is lifelong, particularly in patients with complicated CHD. A proportion of adult patients with CHD, such as those with CoA, TGA, and TOF, may experience late complications, including heart failure and arrhythmias, during their lifetime, despite successful intervention and surgical repair. Consequently, lifetime monitoring and modification of conventional surgery for some complicated CHD are prerequisites to improve the long‐term outcome in patients with CHD.

Fourth, the field of CHD has grown rapidly over the last decade with the application of advanced technology and novel devices. Although the implantation of metal occlusion devices resulted in favorable outcomes in most patients, occluder‐associated complications such as cardiac tissue erosion, residual shunt, arrhythmias, and embolization are not entirely eliminated, which may lead to catastrophic results. In the past decade, several biodegradable occluders have been applied in the treatment of some CHDs, including PFO, ASD, PDA, and VSD in animal experiments and clinical practice.^140^, ^244^ The occluders disintegrated after 6−12 months, achieving complete endothelialization during 12 weeks with fewer complications and no recanalization, which may be superior to metal occluders regarding short‐ and long‐term outcomes.^140^ Nevertheless, the application of biodegradable occluders is still challenging due to their degradation rate, strength, flexibility, and shaping. Moreover, clinical data regarding the long‐term efficacy and safety of biodegradable occluders are limited. More prospective randomized controlled studies with larger numbers of patients are required.

In conclusion, it is still a challenge to determine the suitable timing of intervention and optimal method of treatment in order to better improve the long‐term prognosis of patients with CHD. The management of CHD relies on an improved understanding of the types of CHD, which may facilitate development of personalized therapy. This study reviews the pathophysiology, diagnosis and treatment of CHD (Table 1), aiming to enhance the current understanding of CHD. An effective management strategy can achieve excellent efficacy and lower complications in patients with CHD. As a multidisciplinary care is often necessary to optimize the diagnosis and treatment of patients with CHD, establishing a regional center for screening, diagnosing, and treating CHD with the involvement of cardiologists, pediatrists, obstetricians, radiologists, epidemiologists, and geneticists is essential.

**TABLE 1:** Pathophysiology, diagnosis, and treatment of 13 types of CHD.

CHD types	Incidence	Sex predominance	Genetic risk	Intracardiac shunts	Cyanosis	PAH	HF	Arrhythmia	Diagnosis	Treatment (intervention or surgery)	References
PFO	25% of adult	No	No	R‐L	No	No	No	No	TCD, TTE, TEE	Intervention	14,89
ASD	1.3–2.9/1000 live births	Female	Yes	L‐R (R‐L occurs in case of severe PAH)	Occurs in case of severe PAH	Yes	Yes	Yes	TTE, TEE, CMRI, CCT, ICE	Intervention/surgery	2, 245
VSD	3–3.5/1000	No	Yes	L‐R (R‐L occurs in case of severe PAH)	Occurs in case of severe PAH	Yes	Yes	Yes	TTE, TEE, CMRI	Intervention/surgery	147, 150
AVSD	0.1/1000	No	Yes	L‐R (R‐L occurs in case of severe PAH)	Occurs in case of severe PAH	Yes	Yes	Yes	TTE, CMRI	Surgery	245
PDA	0.9–2/1000	Female	Yes	L‐R (R‐L occurs in case of severe PAH)	Occurs in case of severe PAH	Yes	Yes	Yes	TTE, CMRI, CCT, cardiac catheterization	Intervention/surgery	1, 245, 246
CoA	0.3–0.4/1000	Male	Yes	No	No	Yes	Yes	Yes	TTE, CMRI, CTA,	Intervention/surgery	181, 183, 184
TGA	0.1–0.3/1000	Male	Yes	No	Yes	Yes	Yes	Yes	TTE, CMRI	Surgery	1, 245
ccTGA	Rare	No	Yes	No	Yes	Yes	Yes	Yes	TTE, CMRI	Surgery	1, 245
Coronary anomalies	Rare	No	Yes	No	No	No	Yes	Yes	TTE, CCT, CMRI, Coronary CTA and angiography	Intervention/surgery	210, 215
LVOTO	0.5/1000	No	Yes	No	No	Yes	Yes	Yes	TTE, TEE, CMRI, CCT,CTA, cardiac catheterization	Intervention/surgery	2, 247
RVOTO	0.8/1000	No	Yes	No	Occurs in case of severe pulmonary stenosis	Yes	Yes	Yes	TTE, CMRI, CCT, cardiac catheterization	Intervention/surgery	2
TOF	0.2/1000	No	Yes	R‐L	Yes	Yes	Yes	Yes	TTE, CMRI, CCT	Surgery	245
Ebstein anomaly	Rare	No	Yes	No	Yes	Yes	Yes	Yes	TTE, CMRI	Surgery	240

Abbreviations: ASD, atrial septal defect; AVSD, atrioventricular septal defect; CCT, cardiac computed tomography; ccTGA, congenitally corrected transposition of the great arteries; CHD, congenital heart disease; CMRI, cardiac magnetic resonance imaging; CoA, coarctation of the aorta; CTA, computed tomographic angiography.; HF, heart failure; ICE, intracardiac echocardiography; L‐R, left‐to‐right; LVOTO, left ventricular outflow tract obstruction; PAH, pulmonary arterial hypertension; PDA, patent ductus arteriosus; PFO, patent foramen ovale; R‐L, right‐to‐left; RVOTO, right ventricular outflow tract obstruction; TCD, transcranial doppler; TEE, transesophageal echocardiography; TGA, transposition of the great arteries; TOF, tetralogy of Fallot; TTE, transthoracic echocardiography; VSD, ventricular septal defect.

## AUTHOR CONTRIBUTIONS

X. M., M. S., K. Z., W. D. L., and Y. Y. L. researched the literatures, drafted initial manuscript, and prepared the figures. C. Z. and Y. Z. conceived the study and edited the manuscript. All authors approved the final manuscript for publication.

## CONFLICT OF INTEREST STATEMENT

The authors declare no conflict of interest.

## ETHICS STATEMENT

None applicable.

## Data Availability

Not applicable.
